# New insights into lineage restriction of mammary gland epithelium using parity-identified mammary epithelial cells

**DOI:** 10.1186/bcr3593

**Published:** 2014-01-07

**Authors:** Ted H-T Chang, Kamini Kunasegaran, Gerard A Tarulli, Duvini De Silva, P Mathijs Voorhoeve, Alexandra M Pietersen

**Affiliations:** 1 Program in Cancer & Stem Cell Biology, Duke-NUS Graduate Medical School, 8 College Rd., 169857 Singapore, Singapore; 2 Department of Cellular and Molecular Research, National Cancer Centre Singapore, 11 Hospital Dr., 169610 Singapore, Singapore; 3 Department of Biochemistry, National University of Singapore, 21 Lower Kent Ridge Rd., 119077 Singapore, Singapore; 4 Department of Physiology, National University of Singapore, 21 Lower Kent Ridge Rd., 119077 Singapore, Singapore

## Abstract

**Introduction:**

Parity-identified mammary epithelial cells (PI-MECs) are an interesting cellular subset because they survive involution and are a presumptive target for transformation by human epidermal growth factor receptor 2 (HER2)/neu in mammary tumors. Depending on the type of assay, PI-MECs have been designated lobule-restricted progenitors or multipotent stem/progenitor cells. PI-MECs were reported to be part of the basal population of mammary epithelium based on flow cytometry. We investigated the cellular identity and lineage potential of PI-MECs in intact mammary glands.

**Methods:**

We performed a quantitative and qualitative analysis of the contribution of PI-MECs to mammary epithelial cell lineages in pregnant and involuted mammary glands by immunohistochemistry, fluorescence-activated cells sorting (FACS), and quantitative polymerase chain reaction. PI-MECs were labeled by the activation of Whey Acidic Protein (WAP)-Cre during pregnancy that results in permanent expression of yellow fluorescent protein.

**Results:**

After involution, PI-MECs are present exclusively in the luminal layer of mammary ducts. During pregnancy, PI-MECs contribute to the luminal layer but not the basal layer of alveolar lobules. Strikingly, whereas all luminal estrogen receptor (ER)-negative cells in an alveolus can be derived from PI-MECs, the alveolar ER-positive cells are unlabeled and reminiscent of Notch2-traced L cells. Notably, we observed a significant population of unlabeled alveolar progenitors that resemble PI-MECs based on transcriptional and histological analysis.

**Conclusions:**

Our demonstration that PI-MECs are luminal cells underscores that not only basal cells display multi-lineage potential in transplantation assays. However, the lineage potential of PI-MECs in unperturbed mammary glands is remarkably restricted to luminal ER-negative cells of the secretory alveolar lineage. The identification of an unlabeled but functionally similar population of luminal alveolar progenitor cells raises the question of whether PI-MECs are a unique population or the result of stochastic labeling. Interestingly, even when all luminal ER-negative cells of an alveolus are PI-MEC-derived, the basal cells and hormone-sensing cells are derived from a different source, indicating that cooperative outgrowth of cells from different lineages is common in alveologenesis.

## Introduction

Mammary epithelium is maintained by a pool of stem or progenitor cells that upon asymmetric cell division undergo a process of lineage restriction to generate the different epithelial cell types therein [1,2]. The existence of lineage-restricted cell populations in mammary epithelium has long been recognized [3,4], and because the molecular portraits of breast tumors revealed a clear separation of breast cancer subtypes based on lineage characteristics [5], there has been a concerted effort in recent years to map the mammary stem cell hierarchy [6]. However, the extent of lineage restriction and parent-progeny relationships in this hierarchy are still unclear because most of the data showing multi- or bi-lineage potential has been obtained by isolating single mammary epithelial cells and evaluating their progeny either *in vitro* or by transplantation into de-epithelialized mammary fat pads. For example, several groups have shown that cells belonging to the basal population (based on cell surface markers) have the potential to generate all mammary epithelial cell types when transplanted by themselves in cleared mammary fat pads, and these cells are referred to as mammary stem cells [7-9]. However, lineage-tracing techniques using a basal cell-specific promoter to permanently label basal cells in intact mammary glands showed that these cells gave rise only to basal progeny but not to cells belonging to the luminal layer [10]. Therefore, lineage potential appears to be more restricted in unperturbed tissue.

Another subpopulation of mammary epithelial cells, parity-identified mammary epithelial cells (PI-MECs), has also been shown to have multi-lineage potential in transplantation assays [11]. In the intact mammary gland, however, it is unclear where PI-MECs are positioned in the lineage hierarchy. PI-MECs are an interesting cell population because they are long-lived and have been suggested to be the cancer cell of origin in Her2/neu-driven tumorigenesis [12-14]. PI-MECs are identified by a reporter which is irreversibly activated through Cre-mediated recombination [15]. The Cre recombinase is expressed under control of the whey acidic protein (*WAP*) promoter, which is strongly induced during pregnancy and throughout lactation [16,17]. After weaning, the secretory milk-producing cells are removed during a process termed involution [18] and WAP expression becomes undetectable by Western blot [19]. Unexpectedly, initial studies using WAP-Cre and LacZ-reporter mice showed that not all cells that expressed WAP during lactation are removed, but in fact a significant portion of LacZ^+^ cells survived the involution process [15]. These LacZ^+^ cells were found to be a major source for the newly developing structures required for milk production in subsequent pregnancies [15] and are now referred to as PI-MECs [6].

In the virgin (nulliparous) mouse or in a mouse that has completed the involution process after weaning (parous), the mammary epithelium consists mainly of bi-layered milk ducts [1]. The outer basal layer contains contractile myoepithelial cells as well as the mammary stem cells, and the luminal layer contains both steroid receptor-positive and -negative cells. The steroid receptor-positive cells, or hormone-sensing cells, relay systemic growth signals such as estrogen, progesterone, and prolactin via paracrine signals to neighboring cells, thereby orchestrating dynamic changes in mammary gland morphology [20,21]. Under the influence of pregnancy-induced hormone surges, side branches sprout from the epithelial ducts. On these side branches, lobules of alveoli are formed to become the sites of milk production [20]. LacZ-staining demonstrated a clear contribution of PI-MECs to these alveolar lobules and PI-MECs are therefore also referred to as lobule-limited progenitors [6]. In these analyses based on intact mammary glands, PI-MECs are presumed to generate both the basal and luminal cells that make up the alveoli [6]. Interestingly, transplantation studies of unsorted mammary epithelial cells showed that PI-MECs can generate all mammary epithelial cell types. However, they had a limited capacity for self-renewal when outgrowths consisted of PI-MECs only and therefore they were designated stem/progenitor cells [11]. Fluorescence-activated cell sorting (FACS) studies on cultured primary cells suggested that PI-MECs are part of the basal population [22], in line with the position of the mammary stem cells [8].

In the study presented here, we used a fluorescent reporter gene to analyze the cellular identity and lineage contribution of PI-MECs in unperturbed mammary glands. We demonstrate by both FACS and histology that PI-MECs are steroid receptor-negative luminal cells of the alveolar lineage. Their characteristics are indistinguishable from those of unlabeled alveolar progenitor cells of the same mammary gland. During pregnancy, PI-MECs contribute steroid receptor-negative luminal cells, but not hormone-sensing or basal cells, to developing alveoli.

## Methods

### Mice

WAP-Cre mice (Jax#008735) [23] and Rosa26-lox-Stop-lox-YFP mice [24] were backcrossed up to four generations on an FVB background. All of the mice used in this study contain one allele each of the WAP-Cre and Rosa-lsl-YFP transgenes. For timed matings, female mice were placed in the cage of a male after 5 p.m. and checked for vaginal plugs at 9 a.m. the following morning (day 0). Mice were euthanized by carbon dioxide inhalation and immediately dissected for thoracic (#3), abdominal (#4), and inguinal (#5) mammary glands at the indicated time points. All animal protocols were approved by the SingHealth Institute for Animal Care and Use Committee.

### Imaging and cryosectioning of whole-mounted mammary glands

Thoracic glands were whole mount-photographed on an Olympus SZX-12 fluorescent stereoscopic microscope with green fluorescent protein (GFP) filter (Olympus, Tokyo, Japan), and images were acquired by DP2-BSW software through an Olympus DP72 CCD detector. One #3 gland was subsequently fixed overnight in 4% buffered formaldehyde (ICM Pharma, Singapore) for paraffin embedding. The other #3 gland was fixed 1.5 hours in 2% formaldehyde in phosphate-buffered saline, prior to embedding in Tissue Tek OCT (Sakura Finetek, Tokyo, Japan) for cryopreservation. Cryosections (15 to 30 μm) were cut on a Leica CM1950 cryostat onto SuperFrost Plus-coated slides (Menzel-Gläser, Braunschweig, Germany), and stained with Rhodamine-conjugated phalloidin (Molecular Probes, now part of Invitrogen Corporation, Carlsbad, CA, USA) in accordance with the instructions of the manufacturer. Sections were mounted in Vectashield fluorescence mounting media (Vector Laboratories, Burlingame, CA, USA), and images were acquired on a Zeiss 710 confocal microscope (Carl Zeiss, Jena, Germany) with a pinhole aperture of 1 airy unit.

### Indirect immunofluorescence

Fixed #3 mammary glands were processed and embedded in paraffin wax. Paraffin sections of 5 μm were prepared and subjected to 1 mM disodium-ethylenediaminetetraacetic acid (EDTA) antigen retrieval as described previously [21]. Primary antibodies used for immunofluorescence are the following: cytokeratin-8 (CK8) (TROMA-I, rat, 1:100; Developmental Studies Hybridoma Bank, Iowa City, IA, USA), estrogen receptor (NCL-ER-6 F11, mouse, 1:100; Novocastra, which is part of Leica, Wetzlar, Germany), GFP (600-401-215, rabbit, 1:100; Rockland Immunochemicals Inc., Gilbertsville, PA, USA), GFP (600-141-215, goat coupled to Dylight-488, 1:300; Rockland Immunochemicals Inc.), progesterone receptor (MAB9785, rabbit, 1:400; Abnova, Taipei, Taiwan), and smooth muscle actin (SMA) (A2547, mouse, 1:1,000; Sigma-Aldrich, St. Louis, MO, USA). Secondary antibodies used at 1:400 dilution are from Invitrogen Corporation: Alexa488-coupled goat anti-mouse (A11029), Alexa488-coupled goat anti-rabbit (A11034), Alexa568-coupled goat anti-mouse (A11031), and Alexa568-coupled goat anti-rabbit (A11036). Additionally, CF633nm-coupled donkey anti-rat (20137–1; Biotium, Hayward, CA, USA) was used at 1:400 dilution.

### Isolation of primary mammary epithelial cells for fluorescence-activated cell sorting, RNA, and genomic DNA

Abdominal and inguinal glands were pooled and processed either for single mammary epithelial cell isolation for FACS analysis as previously described [21] or else processed just to the organoid stage for purposes of harvesting RNA and genomic DNA. At this point, three quarters of the organoid suspension was pelleted for lysis in PureZOL RNA isolation reagent (Bio-Rad Laboratories, Inc., Hercules, CA, USA), and total RNA then was prepared in accordance with the instructions of the manufacturer. The remaining quarter was pelleted and resuspended in 0.1 M Tris-Cl pH 8/0.2 M NaCl/5 mM EDTA/0.2% SDS/0.2 mg/mL Proteinase K for overnight digestion at 50°C. Genomic DNA was precipitated out from the digest with 1 volume isopranol, washed in 70% ethanol, and resuspended in 100 μL of water. A hundred nanograms of genomic DNA was amplified per sample, 35 cycles with Taq polymerase (Fermentas, Vilnius, Lithuania).

### Cell labeling, flow cytometric analysis, and fluorescence-activated cell sorting

Fluorochrome-conjugated antibodies were titrated on primary mammary epithelial cells to ensure maximal positive signal-to-background fluorescence ratio. Anti-mouse or anti-rat compensation beads (BD 552843 and 552845, respectively) or both were used for single-stain antibody controls. Compensation controls also included two cellular samples: unstained cells and cells stained with 4′,6-diamidino-2-phenylindole (DAPI) (D8417; Sigma-Aldrich). Cells were incubated with antibodies on ice for 45 minutes with agitation every 15 minutes. Samples were then washed with twice the sample volume and resuspended in L15 (Gibco-Life Technologies, now part of Invitrogen Corporation) with 6% fetal calf serum (FCS) (HyClone, Logan, UT, USA) and 200 ng/mL of DAPI, except non-DAPI compensation controls. All multiple-labeled samples were gated on FSC-A versus SSC-A and doublet discrimination (FSC-H versus FSC-W and SSC-H versus SSC-W) and DAPI negativity. Samples contained anti-CD45 to exclude lymphocytes from analysis. Cells were analyzed and sorted on a BD FACS-Aria II containing 355 nm UV, 488 nm blue, 561 nm yellow-green, and 633 nm red lasers (BD Biosciences, San Jose, CA, USA). Specific antibodies used and gating strategy are detailed in Additional file 1.

### Synthesis of cDNA and quantitative polymerase chain reaction analysis

For analysis of transcript levels by quantitative polymerase chain reaction (qPCR) in FACS-sorted populations, cells were sorted directly into lysis buffer (10 IU RNase inhibitor (Invitrogen Corporation), 2 mM dithiothreitol (DTT), 0.15% Tween-20 (Bio-Rad Laboratories, Inc.) in 12 μL of nuclease-free water) in PCR tubes by using a direct reverse transcription (RT) method described by Ho and colleagues [25]. Five hundred cells were sorted into each tube, and RT was performed by using Superscript VILO (Invitrogen Corporation) in accordance with the protocol of the manufacturer. In the case of PureZOL-purified total RNA harvested from epithelial organoids, cDNA was prepared by using the Bio-Rad iSelect kit (Bio-Rad Laboratories, Inc.) and a pool of gene specific RT primers (each at 0.42 mM final concentration), comprising the reverse primers for each of the amplified genes: *WAP*, *Cre* transgene, *YFP* transgene, *Elf5*, and hypoxanthine-guanine phosphoribosyltransferase (*HPRT*). Primers were designed that span introns to exclude the detection of genomic DNA and selected for optimum melt curve and amplification profiles. The primers to detect the WAP-Cre transgene expression were designed from the Cre sequence and rabbit β-globin polyA sequence which spans the β-globin intron that is spliced out in MECs (data not shown). All primer sequences are listed in Additional file 2. In the case of the intronless YFP transcript, a no-reverse-transcriptase (NRT) control reaction was run in parallel with the normal RT reaction, and qPCR background signal from the NRT reaction (due to genomic DNA contamination in those samples with low RNA yields) was subtracted from the RT reaction quantification for YFP in the reported results. qPCR was performed by using Sso Fast Evagreen supermix reagent (Bio-Rad Laboratories, Inc.) in accordance with the protocol of the manufacturer. To assess genomic recombination of the Rosa-lsl-YFP locus, primer sequences flanking the loxP sites were designed such that the recombined locus could be amplified from organoid genomic DNA as a 578-bp band (Additional file 3), whereas the unrecombined locus would generate a 3,244-bp band (data not shown).

## Results

### Genetic labeling of parity-identified mammary epithelial cells

**Figure 1:**
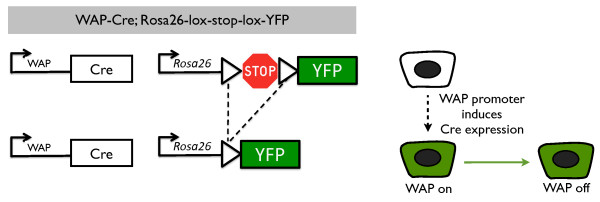
**Labeling of parity-identified mammary epithelial cells (PI-MECs).** In WAP-Cre;Rosa26-lsl-YFP double transgenic mice, yellow fluorescent protein (YFP) is not expressed (white cell) until the whey acidic protein (*WAP*) promoter is induced (by pregnancy). When activation of the *WAP* promoter results in the expression of sufficient levels of the Cre recombinase, the loxP sites (triangles) will be recombined and the sequence in between will be excised (dotted line), bringing the *YFP* gene under control of the constitutively active Rosa26 promoter (green cell, *WAP* on). Upon cell division, daughter cells will remain YFP-positive even though WAP is not expressed (green cell, *WAP* off) as a result of the genetic deletion of the stop sequence.

To investigate the cellular identity and lineage potential of PI-MECs in intact mammary glands, we chose to label them with a fluorescent reporter. The labeling of PI-MECs was first described by Wagner and colleagues [15,23] and makes use of the Cre/lox system to permanently label cells by genetic recombination. They created a transgenic mouse line expressing the Cre recombinase under the control of the *WAP* gene promoter [23]. We crossed these with a reporter strain that contains the *YFP* gene separated from the constitutive Rosa26 promoter by a stop sequence flanked by loxP sites (Rosa26-lox-stop-lox-YFP, or Rosa-lsl-YFP) [24]. Transcriptional activation of the WAP promoter induces expression of Cre recombinase [16], which excises the transcriptional stop sequence in the reporter construct under control of the constitutively active Rosa26 promoter (Figure 1). From that point onwards, the cell and all of its progeny permanently express the *YFP* reporter gene.

**Figure 2:**
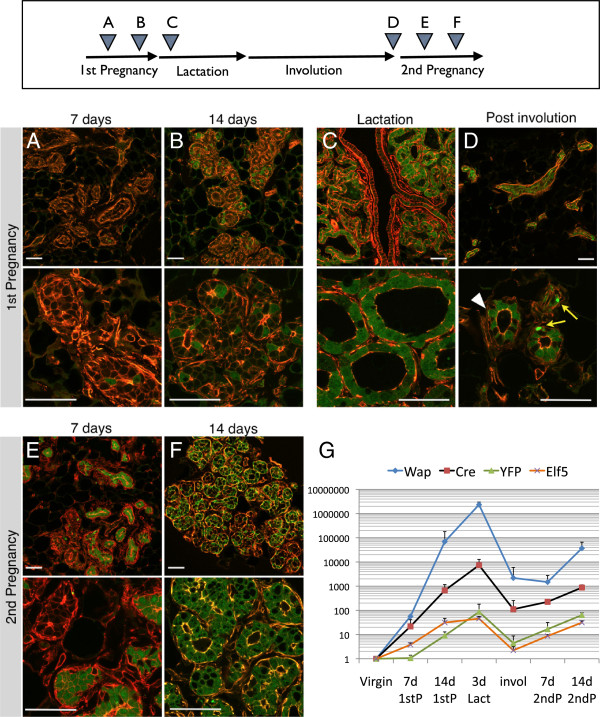
**Analysis of parity-identified mammary epithelial cells (PI-MECs) during first and second pregnancies.** Mammary glands from WAP-Cre; Rosa-lsl-YFP mice were collected at the following stages: 7 days **(A)** and 14 days **(B)** of the first pregnancy, 3 days of lactation **(C)**, involuted (>6 weeks after weaning) **(D)**, and 7 days **(E)** and 14 days **(F)** of the second pregnancy. Cryosections were analyzed for YFP expression (green) and counterstained with rhodamine-conjugated phalloidin (red). Representative confocal microscope images were captured with 20× (upper panels) and 63× (lower panels) objective lenses. Bar is 50 μm. White arrowhead indicates a PI-MEC (YFP^pos^ cell that survived involution), and the yellow arrows indicate collapsed YFP bodies seen often in involuted sections. **(G)** Quantification of relative mRNA levels in mammary epithelial organoid isolates. Gene expression is normalized to hypoxanthine-guanine phosphoribosyltransferase (*HPRT*) expression, and error bars indicate standard deviation of three individual mice at each time point.

We crossed WAP-Cre mice with the Rosa-lsl-YFP strain and collected mammary glands from double transgenic female mice before, during, and after pregnancy (Figure 2). Thoracic mammary glands were examined by wholemount fluorescence microscopy (Additional file 4) and by cryosections (Figure 2) to confirm the proper functioning of the PI-MEC labeling system. The cryosections were counterstained with phalloidin to visualize the alveolar structures independent of their YFP status. At day 7 of the first pregnancy, we detected no evident YFP expression by wholemount analysis (Additional file 4A) and very few YFP-positive (YFP^pos^) cells on cryosections (Figure 2A, see Additional file 5 for close-ups of individual cells). In only one mouse out of five harvested at this stage did we observe extensive YFP labeling of epithelial structures (later determined by FACS to represent approximately 6% of epithelial cells; Additional file 6), which may be attributable to minor alveolar development as part of normal estrus cycling, as previously described [15]. At 14 days of pregnancy, we detected unambiguous activation of the *YFP* reporter gene (Figure 2B), with about half of alveoli containing at least one YFP-expressing cell and a small percentage of alveoli consisting of more than 90% YFP^pos^ cells. This timing precedes reports of labeling using the LacZ reporter, which was undetectable at 14 days and started at only 15 days [11] or 18 days of pregnancy [15]. This could be due to more efficient floxing of the YFP reporter compared with the LacZ reporter or a difference in the sensitivity of detection of the labeled cells. We could detect *WAP* mRNA, and in parallel *Cre* mRNA, in primary MEC preparations at day 7 of the first pregnancy (Figure 2G). Consistent with the observed Cre expression and appearance of more YFP^pos^ cells, we could detect recombination of the genomic Rosa26 locus by PCR at pregnancy day 7 (Additional file 3) which is followed at day 14 by detectable accumulation of mRNA expression for *YFP* (Figure 2G). As expected, *WAP* (and *Cre*) expression reached maximum levels during lactation. Expression of the alveolar specification gene *Elf5*[26] reflects the increase in the number of alveolar cells generated during pregnancy and precedes the appearance of YFP, which is dependent on the accumulation of sufficiently high levels of the Cre recombinase in the individual cells (Figure 2G).

At 3 days post-partum, virtually every alveolus contained only YFP-expressing cells (Figure 2C, see also Additional file 5C), consistent with complete recombination of the Rosa-lsl-YFP locus at this stage and underscoring the efficiency of the WAP-Cre/YFP labeling system. Larger ducts of glands at 3 days lactation often remained negative for YFP, in contrast to secondary ducts or side branches that contain multiple YFP^pos^ cells (Figure 2C, top panel and Additional file 7). Six weeks after weaning, the alveolar structures had been cleared by involution. Only the ductal network and regressed terminal branches remained in the post-involution glands, and YFP^pos^ cells were readily detectable in involuted mammary epithelium (Figure 2D). These YFP^pos^ cells are referred to as PI-MECs, cells present in parous tissue that survived involution. In contrast, a large proportion of cells that were initially labeled during pregnancy terminally differentiated and were removed. Thus, not all YFP^pos^ cells that are present during pregnancy and lactation will become PI-MECs. In parous epithelium, *WAP* and *Cre* expression dropped by several orders of magnitude, consistent with loss of detectable WAP protein at this stage [19]. Notably, the baseline *WAP* mRNA expression remains higher after involution than in virgin mice (Figure 2G), consistent with a recent study from the Bentires-Alj laboratory [27]. Overall, these results were concordant with observations with previously published LacZ and GFP reporters [11,15,22,23,28].

At day 7 of the second pregnancy, alveoli emerged from the involuted ductal network. In contrast to the first pregnancy, some of these contained mostly YFP^pos^ cells, whereas others were unlabeled (Figure 2E). The majority of these YFP^pos^ cells are likely progeny generated by PI-MECs (this will be addressed later). By day 14 of the second pregnancy, most alveoli were completely labeled by YFP (Figure 2F) but with a marked proportion of alveoli that were still partially labeled or even unlabeled. This suggests that, in addition to PI-MEC-derived alveoli which are already YFP-labeled, some alveoli were derived from unlabeled progenitors that progressively undergo *YFP* recombination and activation, perhaps at a somewhat accelerated pace compared with the first pregnancy (compare Figure 2B and F). The increase in cells with a *de novo* recombined YFP reporter coincides with the renewed induction of *WAP* (and in parallel *Cre*) during the second pregnancy (Figure 2G, note the logarithmic scale).

### Parity-identified mammary epithelial cells are luminal cells that express markers of alveolar progenitor cells

**Figure 3:**
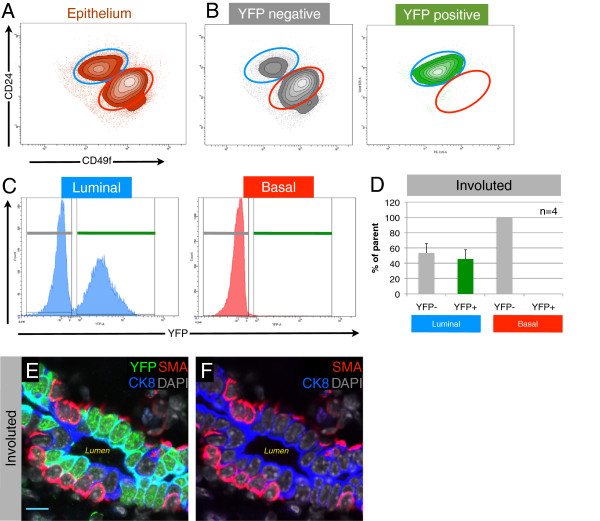
**Parity-identified mammary epithelial cells (PI-MECs) in involuted mammary glands are luminal. (A)** Epithelial cells isolated from involuted mammary glands were labeled with fluorescent antibodies against CD24 and α6-integrin (CD49f) to distinguish the luminal (blue gate) and basal (red gate) population. **(B)** Yellow fluorescent protein-negative (YFP^neg^) cells and YFP^pos^ cells (PI-MECs) are shown separately on density/contour plots. **(C)** The luminal and basal populations are plotted in separate histograms to quantify the proportion of YFP-positive cells in each population **(D)**; error bars indicate standard deviation for 4 individual mice. **(E)** Immunofluorescent probing of involuted mammary epithelium for YFP (PI-MECs, green), the luminal marker cytokeratin-8 (CK8) (blue), and basal marker smooth muscle actin (SMA) (red). Nuclei are labeled by 4′,6-diamidino-2-phenylindole (DAPI) (grey). **(F)** Omission of the YFP signal from the image shown in **(E)** shows the mutual exclusive labeling of luminal cells by CK8 (blue) and basal cells by SMA (red). Bar is 10 μm.

After validation of the reporter system, we set out to characterize the cellular identity of PI-MECs by FACS analysis by using cell surface markers for the various epithelial populations of the mammary gland. Six weeks after weaning, involuted mammary glands were processed to single cells, and luminal and basal MEC populations were identified by staining for CD24 and α6-integrin (CD49f) [21,29], after exclusion of doublets, dead cells, and lymphocytes (Figure 3A and Additional file 1B). Analysis of four individual WAP-cre;Rosa-lsl-YFP animals showed that the YFP^pos^ cells (PI-MECs) fall squarely within the luminal gate (Figure 3B). Plotting the luminal and basal population on separate histograms for YFP further highlights the lack of PI-MECs in the basal population (Figure 3C). Within the luminal population, PI-MECs represent roughly half of the population (Figure 3C and D). Immunofluorescence staining on sections of involuted mammary glands confirmed the exclusive localization of PI-MECs to the luminal layer (Figure 3E and F). Cells identified with an antibody recognizing YFP also expressed the luminal cell-specific marker CK8 but never the basal cell-specific marker SMA. In contrast to the previously reported basal identity of cultured PI-MECs [22], these data definitively establish PI-MECs as a luminal cell type in intact mammary glands.

**Figure 4:**
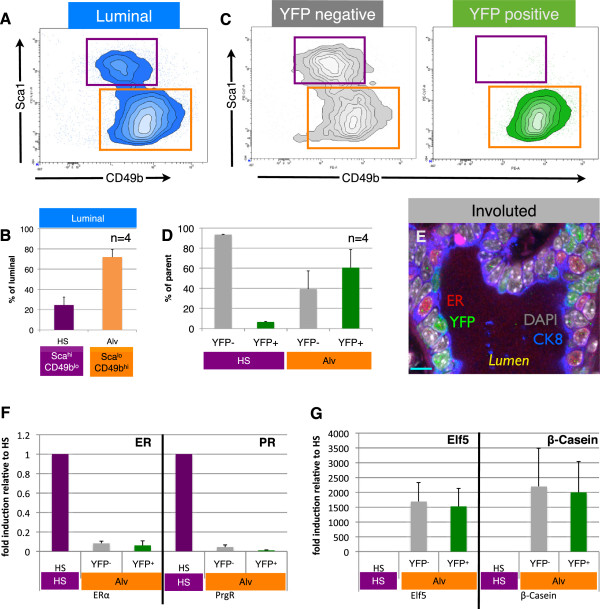
**Parity-identified mammary epithelial cells (PI-MECs) in involuted mammary glands belong to the alveolar progenitor population. (A)** The luminal population of mammary epithelial cells (CD24^hi^ CD49f^lo^) is separated into hormone-sensing cells (Sca1^hi^ CD49b^lo^; purple gate) and alveolar progenitor cells (Sca1^lo^ CD49b^hi^; orange gate). **(B)** Relative proportions of hormone-sensing (HS) cells and alveolar progenitor cells (Alv) within the luminal population. **(C)** Analysis of yellow fluorescent protein-negative (YFP^neg^) (grey) and YFP^pos^ (PI-MEC, green) subpopulations of luminal cells shows that PI-MECs are found mainly in the alveolar progenitor cell population (orange gate). Note that the combined density/contour plot shows the relative distribution of the population on display and therefore the HS population appears larger in **(C)** than in **(A)** because the YFP^pos^ population is plotted separately in **(C)**. **(D)** Distribution of YFP^neg^ cells and YFP^pos^ PI-MECs within populations of HS cells (Sca1^hi^ CD49b^lo^; purple label) and of alveolar progenitor cells (Sca1^lo^ CD49b^hi^; orange label). **(E)** Immunofluorescent probing of an involuted mammary section identifies three luminal cell types: HS cells expressing the estrogen receptor alpha (ERα) (red), PI-MECs expressing YFP (green), and luminal cells expressing CK8 (blue) but neither ER nor YFP. Bar is 10 μm. **(F)** Relative mRNA expression levels of HS cell marker genes *ERα* and progesterone receptor (*PR*) and **(G)** alveolar cell marker genes *Elf5* and *β-Casein* on populations of sorted HS cells (Sca1^hi^CD49b^lo^) or alveolar progenitor cells (Sca1^lo^CD49b^hi^) which were separated based on their YFP expression. Error bars indicate standard deviation for four individual mice.

To further evaluate the cellular identity of PI-MECs within the luminal population, we separated the luminal cells into hormone-sensing cells (Sca1^hi^CD49b^lo^) and estrogen receptor (ER)-negative cells (Sca1^lo^CD49b^hi^) [21,29] (Figure 4A). The ER-negative Sca1^lo^CD49b^hi^ population contains most of the progenitor activity in the luminal population as measured by colony-forming ability [29] and likely contains progenitor cells in different stages of lineage commitment. For clarity, and based on our results described below and the enrichment for markers such as beta-casein and Elf5 [21,30], we refer to the ER-negative Sca1^lo^CD49b^hi^ population as ‘alveolar progenitor cells’.

FACS analysis of MECs isolated from involuted mammary glands and stained for Sca1 and CD49b showed that about a quarter of luminal cells fell within the gate for hormone-sensing cells, whereas the remaining three quarters were found within the alveolar progenitor gate (Figure 4B). YFP-negative (YFP^neg^) cells belonged to both cell types, but the vast majority of YFP^pos^ cells were part of the alveolar progenitor population (Figure 4C). Only around 6% of YFP^pos^ cells were part of the hormone-sensing cell population (Figure 4D). This rare population expresses ERα mRNA to the same extent as the collective hormone-sensing cell population (Additional file 8), suggesting that their FACS profile truly reflects a hormone-sensing identity of these cells. Notably, even though almost all YFP^pos^ cells belonged to the alveolar progenitor population, about 40% of the alveolar progenitor population in the involuted mammary gland lacked YFP expression (Figure 4D). Since secretory alveoli were close to 100% YFP^pos^ at lactation (Figure 2C), this YFP^neg^ population may represent alveolar progenitors that resided in the primary ducts, where most cells are YFP^neg^ even during lactation (Additional file 7). In summary, there appear to be three major populations of luminal cells in involuted mammary epithelium: (a) YFP^neg^ hormone-sensing cells, (b) YFP^neg^ alveolar progenitor cells, and (c) YFP^pos^ alveolar progenitor cells (PI-MECs). Immunofluorescence analysis on tissue sections confirmed the presence of all of these three types of cells and showed their juxtaposition within the luminal layer of ducts in involuted mammary glands (Figure 4E).

We have optimized a direct lysis method for qPCR analysis of limited numbers of cells [25] and used this method to validate the molecular identity of the cell populations identified by FACS. Estrogen receptor alpha (*ERα*) and progesterone receptor (*PR*) expression was largely limited to hormone-sensing cells (Figure 4F), whereas both YFP^pos^ and YFP^neg^ alveolar progenitor populations exclusively expressed the alveolar marker genes *Elf5* and *β-Casein*[26] (Figure 4G). PI-MECs thus clearly belong to the luminal alveolar lineage within the involuted mammary epithelium.

Initially, we performed a microarray on YFP^pos^ and YFP^neg^ luminal cells in order to identify unique cell surface markers of PI-MECs to facilitate future studies of this cell population without the need for generating parous double transgenic mice. However, the presence of a significant proportion of YFP^neg^ alveolar progenitor cells that express *Elf5* and *β-casein* to the same extent as the YFP^pos^ cells explains why we were unable to identify unique markers for PI-MECs. Apart from YFP, which was enriched approximately 30-fold and validates the sorting procedure, no other transcripts were significantly enriched in the YFP^pos^ population (data not shown). This suggests that the YFP^pos^ and YFP^neg^ alveolar progenitor populations share their transcriptome profile beyond *Elf5* and *β-Casein*.

**Figure 5:**
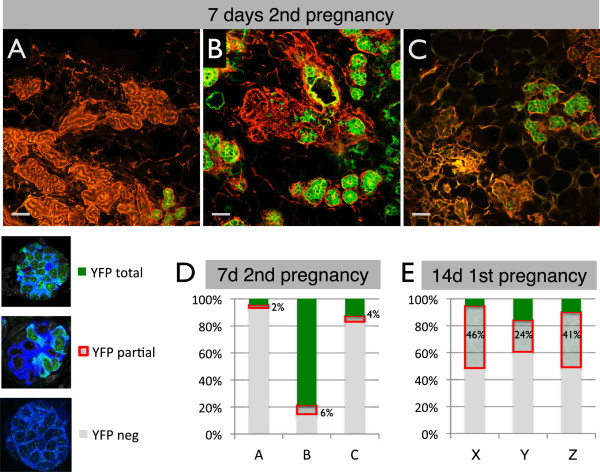
**A large proportion of alveoli derive from non-parity-identified mammary epithelial cells (non-PI-MECs) during the second pregnancy. (A-C)** Cryosections stained with phalloidin (red) of three independent mice used to quantify the relative contribution of PI-MECs to alveolar development at day 7 of the second pregnancy. Scale bar is 50 μm. **(D)** The vast majority of alveoli were scored as either completely yellow fluorescent protein (YFP)-negative (grey) or completely YFP-positive (green), with few alveoli containing a mixture of the two, as highlighted by the red box. **(E)** Quantification of the YFP pattern in alveoli of three mice (X-Z) at day 14 of the first pregnancy (a time of *de novo* floxing). At least 750 alveoli/mouse were counted.

Taken together, our data show that in involuted mammary epithelium apart from PI-MECs there exists an unlabeled alveolar progenitor population with a similar transcriptional and cell surface profile. To address the question of whether this unlabeled alveolar progenitor population is functionally equivalent to PI-MECs, we examined the relative contribution of these populations toward lobuloalveologenesis in a second pregnancy (Figure 5).

### An unlabeled pool of alveolar progenitor cells is equipotent to parity-identified mammary epithelial cells

To evaluate whether PI-MECs or the unlabeled alveolar progenitor cells were the main source for the development of new alveoli, we analyzed WAP-Cre;Rosa-lsl-YFP females at day 7 of their second pregnancy. In typical lineage-tracing techniques, Cre is activated for a short time period following administration of a chemical inducer [31]. In contrast in PI-MEC labeling, *Cre* expression is controlled by WAP promoter activity and therefore the progeny of PI-MECs can be traced only during developmental time periods when WAP is not expressed. We chose the 7-day second pregnancy time point because alveologenesis is already apparent but *WAP* expression is not significantly induced yet ( [15,16] and Figure 2G). Thus, YFP^pos^ alveoli at this time point likely originate from PI-MECs and are not the result of *de novo* activation of the reporter, which occurs again later in the second pregnancy (Figure 2F).

**Figure 6:**
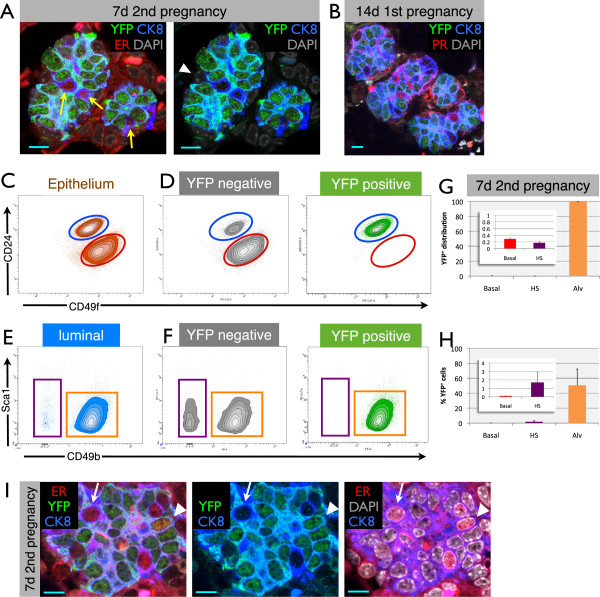
**Parity-identified mammary epithelial cells (PI-MECs) remain lineage-restricted during pregnancy. (A)** Immunofluorescence on paraffin sections from the 7-day second pregnancy time point, showing alveoli in which all cells are yellow fluorescent protein-positive (YFP^pos^), except for rare luminal cells expressing estrogen receptor alpha (ERα) (red, yellow arrows) or basal (CK8-negative, white arrowhead) cells. **(B)** Alveolar clusters at day 14 of the first pregnancy that already consist mostly of YFP^pos^ cells also contain hormone-sensing (HS) cells (labeled by an antibody against the progesterone receptor, red) which do not express YFP. Scale bar is 10 μm. **(C)** Mammary epithelial cells from 7-day pregnant glands (second pregnancy) labeled with CD24 and CD49f to segregate luminal (blue gate) from basal (red gate) single-cell populations. **(D)** YFP^neg^ cells and YFP^pos^ cells (PI-MECs) are shown separately on density/contour plots for CD24 and CD49f. **(E)** Sca1 and CD49b plots of the luminal cell population at 7 days of pregnancy, showing the HS cell population (purple gate) and alveolar cells (orange gate). **(F)** Distribution of YFP^neg^ cells and YFP^pos^ PI-MECs within populations of HS cells and of alveolar cells. Note that the combined density/contour plot shows the relative distribution of the population on display and therefore the HS population appears larger in **(D)** than in **(C)** because the YFP^pos^ population is plotted separately in **(D)**. **(G)** Distribution of YFP^pos^ cells across the different populations. Inset: magnified basal and HS cell bars. **(H)** Percentage of YFP^pos^ cells observed within the basal, HS, and alveolar cell populations. Error bars indicate standard deviation of four independent mice. **(I)** A small percentage of ER^+^ cells are also YFP^pos^ (arrowhead), although most HS cells are YFP^neg^ (arrow). Scale bar is 10 μm.

In involuted glands, roughly half of the alveolar progenitor population was YFP^pos^ (Figure 4D). Strikingly, at day 7 of the second pregnancy, newly forming alveoli stained for YFP either almost completely or not at all (Figure 2E and Figure 5A-C, more examples in Additional file 9). Therefore, besides the PI-MECs that give rise to YFP^pos^ alveoli, there is a significant contribution of unlabeled cells that can form morphologically indistinguishable alveoli. Notably, the number of alveoli derived from PI-MECs or unlabeled alveolar progenitor cells varied widely between animals. To quantify this effect, a cryosection of an entire mammary gland from each of the three mice was scanned on a confocal microscope and reconstituted digitally (see Additional file 10 for an example). Each individual alveolus from a section was then scored into one of three categories: YFP^neg^, YFP^pos^, or partially labeled by YFP. To be considered YFP^pos^, at least 90% of the cells in the alveolus must express YFP, since we had noticed that alveoli frequently contain a few ER-positive hormone-sensing cells which are YFP^neg^ (Figure 6). In all three 7-day pregnant samples, the vast majority of alveoli were scored as either YFP^pos^ or YFP^neg^ (Figure 5A-D and Additional file 11). This all-or-nothing distribution suggests a clonal contribution from either an unlabeled alveolar progenitor or a PI-MEC rather than a mixture of alveolar progenitors contributing to the same alveolus. The small proportion of alveoli that had a mixture of YFP^pos^ and YFP^neg^ cells could signify the mixed contribution of a labeled and an unlabeled progenitor, but these partially labeled alveoli could also reflect early *de novo* activation of the reporter. Indeed, reporter activation due to induction of *WAP* expression at 14 days of the first pregnancy shows a similar stochastic pattern and the majority of alveoli that become labeled at this point belong to the YFP^partial^ category (Figure 5E and Additional file 11). Together with the radically different ratio of partial-versus-totally labeled alveoli (1:3 at day 7 of the second pregnancy compared with 5:1 at day 14 of the first pregnancy), these data fit best with a model whereby at day 7 of the second pregnancy the frequency of *de novo* floxing is still low and the majority of YFP^pos^ cells are generated by PI-MECs.

Overall, the relative contribution of PI-MECs to alveologenesis, as measured by the fraction of total YFP^pos^ alveoli, varied from 4% to 79%. A similar variation was found when four independent animals were analyzed by FACS (27% to 77%, Figure 6H). It is currently unclear whether this variation is stochastic or whether certain conditions favor one population over the other. It should be noted, however, that the females in Figure 5A and B, which show the most divergence in PI-MEC contribution, are littermates who shared nearly identical histories. The proportion of YFP^pos^ alveoli was roughly similar in the right and left mammary glands of each individual mouse (data not shown). This suggests that the relative contribution of PI-MECs and unlabeled alveolar progenitors to alveologenesis is due to systemic regulation rather than independent gland-autonomous effects.

In summary, even though PI-MECs contribute significantly to the generation of new alveoli, a substantial proportion of newly developing alveoli are unlabeled. Therefore, the unlabeled alveolar progenitor cells that are present after involution not only are very similar to PI-MECs in their transcriptome and cell surface markers but also have the ability to contribute to alveologenesis to the same extent as PI-MECs.

### Basal and hormone-sensing cells of developing alveoli are not generated by parity-identified mammary epithelial cells

**Figure 7:**
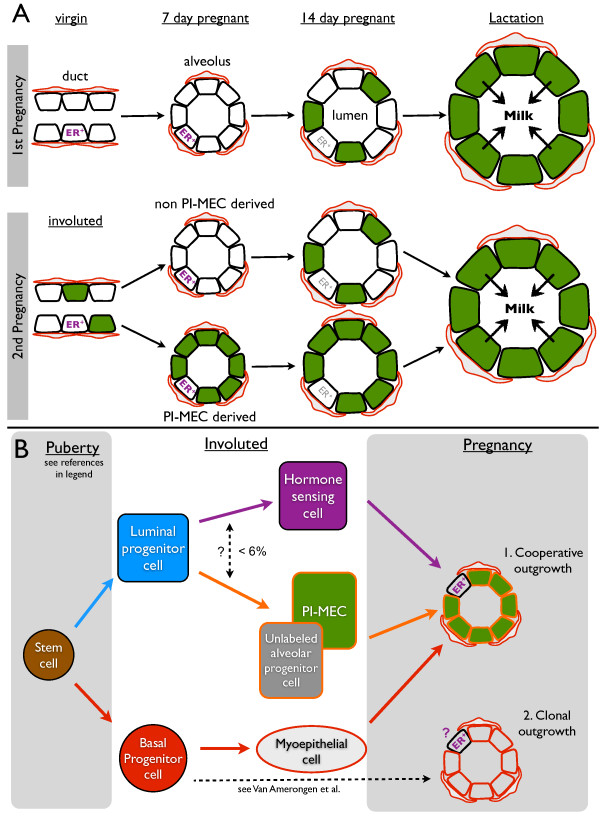
**PI-MEC lineage tracing (A) and a stem cell hierarchy for intact mammary glands (B). (A)** The YFP reporter becomes robustly activated by 14 days of the first pregnancy (green) and by lactation virtually all secretory cells are labeled. After involution, labeled cells are present in the luminal layer of the ducts (only stage where PI-MECs can be definitively recognized, black diamond). At 7 days of the second pregnancy, PI-MECs give rise to all of the ER-negative luminal cells of some but not all alveoli (*WAP* promoter activity is low at this stage). PI-MECs do not generate the alveolar basal cells (red) and ER+ cells (purple). By 14 days of the second pregnancy, the *WAP* promoter is significantly induced, and ER-negative luminal cells in alveoli become labeled *de novo*. ER expression becomes more difficult to detect (grey), but the hormone-sensing cells still express PR and not YFP (see Figure 6). **(B)** Bipotent stem cell activity was reported during embryonic development and puberty [10,34]. In adult non-pregnant mammary glands, unipotent cells maintain the luminal and basal layer [10,34]. We show that PI-MECs are located in the luminal layer and belong to the alveolar lineage (orange). This lineage also contains a population of unlabeled cells with a similar cell surface and gene expression profile. A small proportion of ER+ cells is also YFP^pos^ (< 6%) but it is unclear whether this is due to lineage plasticity or a technical artifact. During pregnancy, a significant proportion of alveoli is formed by cooperative outgrowth of ER-negative luminal cells , hormone-sensing cells and basal cells (7B-1, see Figure 6 and Discussion). An unknown proportion of alveoli contain luminal and basal cells derived from the same Axin2-traced basal progenitor [34] (7B-2), it remains to be tested if this progenitor also generates hormone-sensing cells.

At low magnification, clusters of alveoli appeared to be clonally derived from PI-MECs (for example, Figure 5B and Additional file 9); however, at a higher magnification, it became apparent that even though the majority of cells in these alveoli were YFP^pos^, they consistently contained a small fraction of YFP^neg^ cells. To investigate the cellular identity of these YFP^neg^ cells, we stained paraffin sections with antibodies against YFP, ER, and CK8. Figure 6A shows a typical example from the 7-day second pregnancy time point, whereby all luminal cells in an alveolus are YFP^pos^, except the few cells that express the ER (in red) (more examples in Additional file 12). CK8-negative basal cells are also YFP^neg^, like hormone-sensing cells (Figure 6A). The small proportion of alveoli that are almost fully recombined at 14 days of the first pregnancy also contain a low proportion of YFP^neg^ cells (Figure 6B). We noted that the signal intensity for ER is already reduced by 7 days of pregnancy and becomes virtually undetectable at 14 days, and therefore we used an antibody against progesterone receptor for sections from 14-day pregnant animals. Again, the majority of YFP^neg^ cells are hormone-sensing cells (Figure 6B), similar to the 7-day second pregnancy time point (Figure 6A). Thus, even though all luminal alveolar cells derive from a common progenitor cell (based on the clonal appearance at 7 days of the second pregnancy), developing alveoli contain cells from different lineages; PI-MECs are able to contribute all ER-negative luminal cells to a cluster of alveoli, but the hormone-sensing and basal cells are derived from different lineages (see Figure 7 for a schematic representation of these observations).

Since PI-MECs can give rise to all mammary epithelial cell types in transplantation experiments [11,15,22], we further evaluated their lineage potential *in vivo* during a normal pregnancy by FACS analysis (Figure 6C). Separating the mammary epithelial population of WAP-Cre;Rosa-lsl-YFP mice at 7 days of the second pregnancy into YFP^neg^ and YFP^pos^ subpopulations showed that the majority of YFP^pos^ cells remained restricted to the luminal cell population (Figure 6D). Only 0.29% ± 0.03% of PI-MEC-derived cells were found in the basal cell compartment, indicating that PI-MECs cannot be the source of the expansion of the basal layer during pregnancy. A closer examination of the luminal subpopulations showed that during pregnancy the hormone-sensing cells lose their CD49b and especially their Sca1 expression (Figure 6E versus Figure 4A), consistent with a previous report [29]. The cellular identity of the FACS populations was again validated by qPCR. Even during pregnancy, more than 98% of PI-MECs remained firmly within the luminal alveolar progenitor population (Figure 6F and G). These data show that, as a rule, PI-MECs remain restricted to the luminal alveolar lineage during pregnancy and do not contribute significantly to the other lineages.

Out of all the hormone-sensing cells analyzed by FACS at the 7-day second pregnancy time point, only 1.7% ± 1.2% were YFP^pos^ (Figure 6H). Even though the percentage of YFP^pos^ cells in the hormone-sensing cell gate was small, it is not noise in the FACS data, because rare cells that were positive for both ER and YFP were detectable by confocal microscopy of tissue sections from pregnant mice (Figure 6I).

Taking these findings as a whole, we conclude that, in contrast to the clear multi-lineage potential of PI-MECs in transplantation assays, the lineage potential of PI-MECs *in vivo* is almost entirely restricted to luminal ER-negative cells, even during pregnancy.

## Discussion

### Parity-identified mammary epithelial cells are restricted luminal estrogen receptor-negative progenitors

We show that PI-MECs are a strictly luminal population, both by FACS analysis (including qPCR validation of the different populations) and by confocal microscopy on tissue sections from primiparous and pregnant mice. Matulka and colleagues [22] reported that PI-MECs were located at the tip of the basal cloud, the same position as the mammary stem cells. They used a GFP reporter strain to label the PI-MECs and made use of the same cell surface markers (CD24 and CD49f), but an important difference is that the cells were cultured for 3 days before FACS analysis. Based on the reproducibility of our results in all animals examined and the agreement between different methods, we conclude that PI-MECs are a luminal cell type.

In line with the labeling based on expression of one of the milk genes (*WAP*), we found that PI-MECs have an alveolar identity, based on cell surface expression (Sca1^lo^CD49f^hi^) and gene expression (*Elf5*, *β-casein*). Consistent with previous reports, PI-MECs contribute significantly to alveolar development in pregnancy, and we therefore designate them luminal alveolar progenitor cells.

Unexpectedly, we noted that, at least in primiparous mice, there is a population of YFP^neg^ alveolar progenitor cells that displays characteristics similar to those of PI-MECs. Possibly, these cells resided in the larger ducts during lactation where hardly any cells were YFP-labeled, in contrast to the almost complete labeling of the secretory cells in the alveoli (Additional file 7). Interestingly, there seemed to be a gradient of reporter activation in the ducts because we detected quite a number of YFP^pos^ cells in the smaller ducts of lactating glands. This raises the question of whether PI-MECs might be part of an alveolar progenitor reservoir that is located in the ducts rather than representing fully committed secretory cells that dedifferentiated and reintegrated into the ducts during involution, but a different experimental system is required to address this.

The use of the *WAP* promoter to label PI-MECs creates a technical challenge to investigate PI-MEC progeny, because new YFP^pos^ cells could be either daughter cells of PI-MECs or the result of activation of the *WAP* promoter. Even though we found a general increase in baseline activity of the *WAP* promoter in parous MECs (similar to [27]) and in early pregnancy, our data indicated that *de novo* floxing activity was low at day 7 of the second pregnancy and we used this time point to analyze the lineage potential of PI-MECs. We used both FACS and confocal microscopy to analyze the progeny generated by the YFP-labeled alveolar progenitors in early pregnancy and found that PI-MECs can produce all the steroid receptor-negative luminal cells of an alveolus; however, as a rule, they do not generate basal or hormone-sensing cells of alveoli (Figure 7A). The more restricted lineage potential observed in recent lineage-tracing strategies based on intact mammary glands compared with previous transplantation studies has prompted the revision of current models of the mammary stem cell hierarchy [10,32]. Previously, PI-MECs have been designated (limited) stem cells based on their contribution to multiple lineages in cleared fat pad transplantations [11,14,32]. The data presented here warrant a revision of the place of PI-MECs in the hierarchy: strictly into the luminal lineage (Figure 7B). Note that our observation that PI-MECs generate luminal but not basal cells is consistent with a study showing that in intact mammary glands basal cells maintain the basal layer and luminal cells maintain the luminal layer [10].

Even though it is clear that PI-MECs are remarkably restricted to the luminal alveolar lineage, we observed a small percentage of labeled cells that belonged to the luminal ER^+^ population by FACS and in tissue sections of early pregnancy. It remains to be determined whether this reflects a low level of plasticity of PI-MECs *in situ* (Figure 7B) or whether hormone-sensing cells induce *WAP* expression in particular circumstances. Interestingly, the multipotency of PI-MECs in reconstitution assays suggests that the conditions of transplantation can unlock their lineage restriction. However, cells from the basal layer have the most robust long-term multipotent potential in transplantation assays [9,33], whereas the reconstitution potential of PI-MECs or luminal populations identified by other methods is more limited [11,33], suggesting that some lineage restriction remains even in transplantation assays. It will be interesting to investigate what factors control the lineage potential of these different cell populations both in intact mammary glands and in reconstitution assays and to what extent transplant conditions reflect circumstances relevant for tumorigenesis and metastasis.

### Alveoli are formed by collaborating cell types

An alternative version of the mammary stem cell hierarchy has been proposed on the basis of transplantation experiments that show ductal-limited and lobule-limited mammary epithelial outgrowths [3]. Instead of a division into a basal and a luminal lineage as discussed above [32], this alternative model proposes that stem cells give rise to a ductal- and lobule-restricted lineage whereby the lobule-restricted progenitors are thought to give rise to both the basal and luminal cells of alveoli [6,13]. We did not detect the activity of a bipotent lobule-restricted progenitor, because we did not observe any YFP^pos^ basal cells by histology, and less than 0.1% of basal cells were YFP^pos^ by FACS analysis and could be technical noise. Consistent with our observation that the basal cells in alveoli are derived from a different source are recent lineage-tracing experiments with the *Axin2*-promoter whereby Cre-ER was activated briefly before puberty. In those experiments, clonal Axin2-traced offspring contributed exclusively to the basal layer of multiple clusters of alveoli [34]. In contrast, when Cre activity was briefly induced in adult females, the authors found evidence that Axin2-traced progeny could contribute to both the luminal and basal layers of alveoli [34] (Figure 7B). Interestingly, after involution, Axin2-traced cells remained in the luminal layer, suggesting that PI-MECs could potentially be generated from Axin2-traced basal cells. The Axin2-lineage tracing experiments demonstrate that bipotent activity can be detected in intact mammary glands of adult mice; however, it is unclear how common this activity is. Our data show that PI-MECs make a significant, though variable, contribution to alveologenesis, but this does not include basal alveolar cells.

Moreover, PI-MECs generally do not generate the luminal ER-positive cells, even when all ER-negative luminal cells of an alveolus are derived from PI-MECs. Interestingly, lineage-tracing of cells that expressed *Notch2* in puberty revealed two previously unrecognized cell types—small string cells and large L cells—and these cell types do not fit in current models of the stem cell hierarchy [35]. The cells from the Notch2-lineage are functionally required for alveologenesis, and the authors showed that each alveolus contains a small proportion of L cells. An inverse picture of what we have observed was found in Notch2-lineage tracing experiments in early pregnancy: alveoli in which only a single L cell is labeled and the remaining luminal alveolar cells are unlabeled and thus derived from a different source [35]. In addition, hormone-sensing cells play an important role in the early stages of alveologenesis due to their secretion of paracrine factors such as receptor activator of nuclear factor kappa-B ligand (RANKL) and insulin-like growth factor 2 (IGF-2) [21,36-38]. Together, these observations warrant the investigation of steroid-receptor status of Notch2-lineage traced L cells, and if they prove to be ER-positive, they could be incorporated into the hormone-sensing lineage of the stem cell hierarchy.

Overall, the Notch2-lineage tracing data and the experiments presented here highlight that, as a rule, alveoli develop through collaboration of different cell types (Figure 7B-1) rather than being generated as clonal units from a single progenitor. In the case of cooperative growth, basal cells generate the basal cells of alveoli and luminal alveolar progenitors such as PI-MECs produce luminal ER-negative cells. Hormone-sensing cells may be recruited in small numbers from the ducts and are the likely instigators of alveolar outgrowth [20,21]. The Axin2-lineage tracing data suggest that under certain circumstances alveolar luminal and basal cells can derive from the same source (Figure 7B-2). It will be interesting to determine whether this includes ER-positive cells and how these different modes for alveologenesis, collaboration versus clonal origin, are regulated.

## Conclusions

In intact mammary glands, PI-MECs are strictly luminal and express markers of the secretory alveolar lineage. Notably, an equipotent population of unlabeled alveolar progenitors exists in primiparous glands, raising the question of whether PI-MECs are a unique population or the result of stochastic reporter activation. In line with other studies that showed a more restricted lineage potential of other stem/progenitor populations in intact mammary glands compared with transplantation assays, we found that the potential of PI-MECs *in vivo* is restricted to luminal steroid receptor-negative cells. Our data underscore that individual alveoli are not necessarily clonal and are frequently formed by cooperative outgrowth of cells from different lineages, including at least one basal cell, one ER-positive hormone-sensing cell, and one restricted luminal alveolar progenitor cell such as a PI-MEC.

## Abbreviations

CK8: cytokeratin-8; DAPI: 4′,6-diamidino-2-phenylindole; EDTA: ethylenediaminetetraacetic acid; Elf5: E74-like factor 5; ER: estrogen receptor; FACS: fluorescence-activated cell sorting; FSC: forward scatter; GFP: green fluorescent protein; HER2: human epidermal growth factor receptor 2; HPRT: hypoxanthine-guanine phosphoribosyltransferase; MEC: mammary epithelial cell; NRT: no reverse transcriptase; PI-MEC: parity-identified mammary epithelial cell; PR: progesterone receptor; qPCR: quantitative polymerase chain reaction; Rosa-lsl-YFP: Rosa26-lox-Stop-lox-YFP; RT: reverse transcriptase; SD: standard deviation; SDS: sodium dodecyl sulfate; SMA: smooth muscle actin; SSC: side scatter; WAP: whey-acidic protein; YFP: yellow fluorescent protein; YFPneg: yellow fluorescent protein-negative; YFPpos: yellow fluorescent protein-positive.

## Competing interests

The authors declare they do not have competing interests.

## Authors’ contributions

AP helped to conceive of the hypothesis, to write the manuscript, and to direct the project. GT helped to conceive of the hypothesis and to collect tissues and process them for microscopy. MV helped to conceive of the hypothesis and to direct the project and revised the manuscript. TC helped to collect tissues and process them for microscopy, helped to analyze samples, isolated organoids and performed the molecular analyses, and helped to write the manuscript. KK helped to collect tissues and process them for microscopy, to analyze samples, and to isolate and sort single cells by FACS for qPCR analysis. DS helped to collect tissues and process them for microscopy and helped to isolate and sort single cells by FACS for qPCR analysis. All authors read and approved the final manuscript.

## Supplementary Material

Additional file 1 **Fluorescence-activated cell sorting (FACS) antibodies used and gating strategy. (A)** Antibodies used in FACS isolation to separate luminal and basal populations (without Sca1) and additionally separate hormone-sensing and alveolar populations (with Sca1) of mammary epithelial cells. **(B)** Gating strategy for FACS analysis and sorting. Click here for file

Additional file 2 **Polymerase chain reaction (PCR) primers used for gene expression quantification by quantitative PCR (qPCR) and RosaYFP locus recombination detection.** YFP, yellow fluorescent protein. Click here for file

Additional file 3 **Recombination of Rosa-lsl-YFP locus in pregnant WapCre;Rosa-lsl-YFP glands.** Genomic DNA was harvested from mammary epithelial organoids from double transgenic mice (WapCre;Rosa-lsl-YFP) at the indicated developmental time points. Polymerase chain reaction (PCR) primers flanking the loxP sites of the Rosa26-lox-Stop-lox-YFP locus amplify a 578-bp band only following Cre-mediated recombination. The excision of the stop sequence was detectable at 7 days of the first pregnancy, although the extent of recombination was variable between animals. Rosa-lsl-YFP, Rosa26-lox-Stop-lox-yellow fluorescent protein. Click here for file

Additional file 4 **Analysis of parity-identified mammary epithelial cell (PI-MEC) labeling in sequential pregnancies by wholemount stereoscopy.** Mouse thoracic mammary glands were dissected from WAP-Cre;Rosa-lsl-YFP double transgenic mice at the following stages: 7 days **(A)** and 14 days **(B)** of the first pregnancy, 3 days of lactation **(C)**, involuted (>6 weeks after weaning) **(D)**, and 7 days **(E)** and 14 days **(F)** of the second pregnancy. Yellow fluorescent protein (YFP) fluorescence is visualized by wholemount microscopy of unfixed mammary glands. Bar is 1 mm. Click here for file

Additional file 5 **Parity-identified mammary epithelial cell (PI-MEC) cryosections close up.** Close-up views of the high-magnification confocal images from Figure 2, showing yellow fluorescent protein (YFP) fluorescence (green) from cryosections counterstained with rhodamine-conjugated phalloidin (red) and 4′,6-diamidino-2-phenylindole (DAPI) (grey). WAP-Cre;Rosa-lsl-YFP mouse mammary glands were harvested at 7 days **(A)** and 14 days **(B)** of the first pregnancy, at 3 days of lactation **(C)**, at 6 weeks post-involution **(D)**, and at 7 days **(E)** and 14 days **(F)** of the second pregnancy. Scale bar is 10 μm. Rosa-lsl-YFP, Rosa26-lox-Stop-lox-yellow fluorescent protein. Click here for file

Additional file 6 **Day 7 first pregnant mammary gland with elevated parity-identified mammary epithelial cell (PI-MEC) labeling.** One day 7 first pregnant WapCre; RosaYFP mouse out of five displayed extensive recombination and yellow fluorescent protein (YFP) expression throughout ductal and alveolar network, seen by **(A)** wholemount fluorescence of a thoracic gland and **(B)** fluorescence-activated cell sorting (FACS) histogram of YFP fluorescence of mammary epithelial cells from pooled abdominal and inguinal glands. Bar is 1 mm. Click here for file

Additional file 7 **Ductal labeling in a lactating gland. Enlarged section of top panel of Figure **2**C.** Cryosection of a mammary gland obtained 3 days post-partum from a WAP-Cre;Rosa-lsl-YFP mouse showing yellow fluorescent protein (YFP) expression (green) and counterstained with rhodamine-labeled phalloidin (red). Representative confocal microscope image captured with a 20× objective lens. Arrows point to unlabeled ductal cells and arrowheads to YFP-labeled ductal cells. Scale bar is 50 μm. Click here for file

Additional file 8 **Quantitative polymerase chain reaction (qPCR) identity of estrogen receptor-positive/yellow fluorescent protein-positive (ER**^**+**^**/YFP**^**+**^**) double-positive cells in primiparous mammary epithelial cells (MECs).** Luminal single cells were isolated from parous WapCre;Rosa-lsl-YFP mammary glands and sorted directly into lysis buffer before reverse transcriptase (RT) and qPCR. Rare YFP^pos^ hormone-sensing (HS) (Sca1^hi^ CD49b^lo^) cells (boxed in red) express similar levels of *ER*α and progesterone receptor (*PR*) as YFP^neg^ HS cells and do not express alveolar marker genes *Elf5* or *β-Casein*. Error bars reflect standard deviation (SD) for alveolar samples of 500 cells from three individual mice. Note that the HS data represent one tube of 500 cells from a pooled sample of five mice. Click here for file

Additional file 9 **Contribution of parity-identified mammary epithelial cells (PI-MECs) to alveologenesis occurs in clusters.** Cryosections of mammary glands from WAP-Cre;Rosa-lsl-YFP mice at day 7 of the second pregnancy showing yellow fluorescent protein (YFP) expression (green) counterstained with rhodamine-conjugated phalloidin (red). Grey bar is 50 μm. Click here for file

Additional file 10 **Alveolar section map of a mammary gland at day 7 of the second pregnancy.** A cryosection of a thoracic mammary gland was stained with rhodamine-conjugated phalloidin, imaged by confocal microscopy in overlapping fields covering all alveoli within the section, assembled into a contiguous map with Adobe Photoshop (Adobe Systems, Mountain View, CA, USA), and scored for yellow fluorescent protein (YFP) expression per alveolus. This mouse mammary gland represents Figure 5B. Bar is 1 mm. Click here for file

Additional file 11 **Quantification of yellow fluorescent protein (YFP)-positive alveoli. (A)** Summary of tabulated raw alveoli counts for mice scored in Figure 5D (at day 7 of the second pregnancy, A-B) and for three mice at day 14 of their first pregnancy (X-Z, Figure 5E). **(B)** Confocal images for mice X-Z showing the mainly partial labeling of alveoli at this stage. Scale bar is 50 μm. Click here for file

Additional file 12 **Parity-identified mammary epithelial cells (PI-MECs) generally do not generate hormone-sensing cells.** Representative confocal image of immunofluorescent staining of a paraffin section from a day 7 second pregnancy gland showing a region where all luminal (cytokeratin-8-positive, or CK8^+^, blue) estrogen receptor (ER)-negative cells are derived from PI-MECs (green). In the same alveoli, the luminal (CK8^+^, blue) cells that express the ER (ER^+^, red) are all yellow fluorescent protein-negative (YFP^neg^). Scale bar is 10 μm. Click here for file
