# Supplementary material for: New insights into lineage restriction of mammary gland epithelium using parity-identified mammary epithelial cells
Source: Breast Cancer Res. 2014 Jan 7;16(1):R1. doi: 10.1186/bcr3593 (PMC3978646; doi:10.1186/bcr3593)

A

FACS antibody	species	vol per 250µl	Supplier	catalog number	fluorophore
**CD24**	rat	6µl	eBioscience	93-0242-41	eFluor 605NC
**CD49b**	hamster	1µl	Becton Dickinson	558759	PE
**CD49f**	rat	0.5µl	Becton Dickinson	551129	PE-Cy5
**CD45**	rat	0.5µl	Becton Dickinson	550944	Per CP-Cy5.5
**Sca1**	rat	1.5µl	Becton Dickinson	558162	PE-Cy7

B

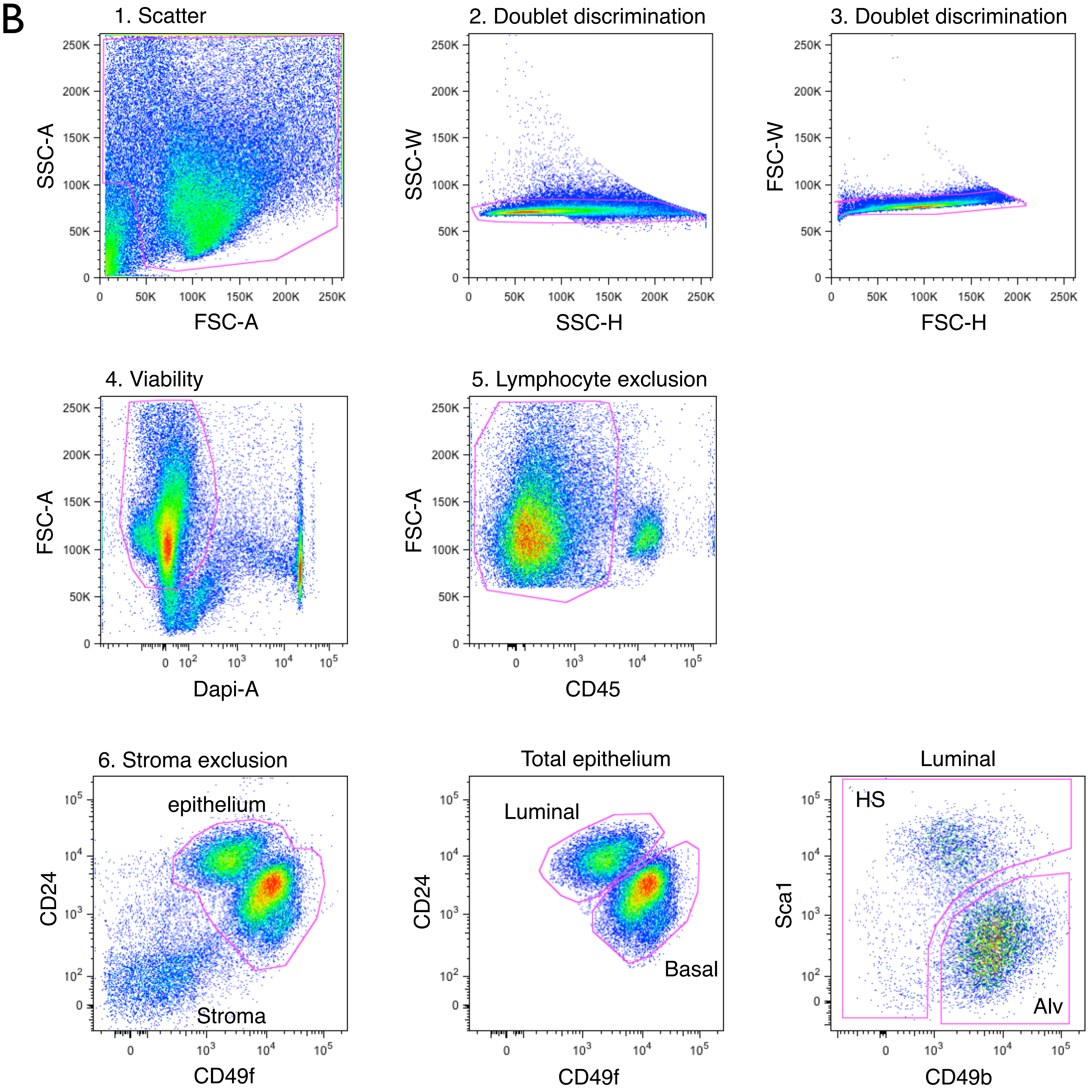

Supplement: Additional file 1 — Fluorescence-activated cell sorting (FACS) antibodies used and gating strategy. (A) Antibodies used in FACS isolation to separate luminal and basal populations (without Sca1) and additionally separate hormone-sensing and alveolar populations (with Sca1) of mammary epithelial cells. (B) Gating strategy for FACS analysis and sorting. [file bcr3593-S1.pdf]
