# Supplementary material for: New insights into lineage restriction of mammary gland epithelium using parity-identified mammary epithelial cells
Source: Breast Cancer Res. 2014 Jan 7;16(1):R1. doi: 10.1186/bcr3593 (PMC3978646; doi:10.1186/bcr3593)
Supplement: Additional file 2 — Polymerase chain reaction (PCR) primers used for gene expression quantification by quantitative PCR (qPCR) and RosaYFP locus recombination detection. YFP, yellow fluorescent protein. [file bcr3593-S2.pdf]

Chang_Additional File 2

Gene	Accession Number	Forward primer	Reverse primer
Estrogen receptor alpha (ESR1)	NM_007956.4	GCCAAGGAGACTCGCTACTG	CTCCGGTTCTTGTCAATGGT
Progesterone receptor (PgR)	NM_008829.2	GGTGGAGGTCGTACAAGCAT	CTCATGGGTCACCTGGAGTT
Elf5	NM_010125.3	GGACTCCGTAACCCATAGCA	TACTGGTCGCAGCAGAATTG
β-Casein (Csn2)	NM_009972	TCCACAACATTCCGTTTCTG	AGCATGATCCAAAGGTGAAAA
HPRT	NM_013556	CTGGTGAAAAGGACCTCTCG	TGAAGTACTCATTATAGTCAAGGGCA
WAP	NM_011709.5	GCCCAATGAAGATAGAATGCTC	GGCTGCTCACTGAAGGGTTA
WAP-Cre transgene	NA	TAGTGAAACAGGGGCAATGG	GACAGCACAACAACCAGCAC
RosaYFP for qPCR	NA	GTGGATCCGGAACCCTTAAT	GTCGACGGTATCGATAAGCTA
RosaYFP (genomic 578bp)	NA	CTTCCCTCGTGATCTGCAAC	AAGTCGTGCTGCTTCATGTG
