# Supplementary figures and images for: New insights into lineage restriction of mammary gland epithelium using parity-identified mammary epithelial cells
Source: Breast Cancer Res. 2014 Jan 7;16(1):R1. doi: 10.1186/bcr3593 (PMC3978646; doi:10.1186/bcr3593)

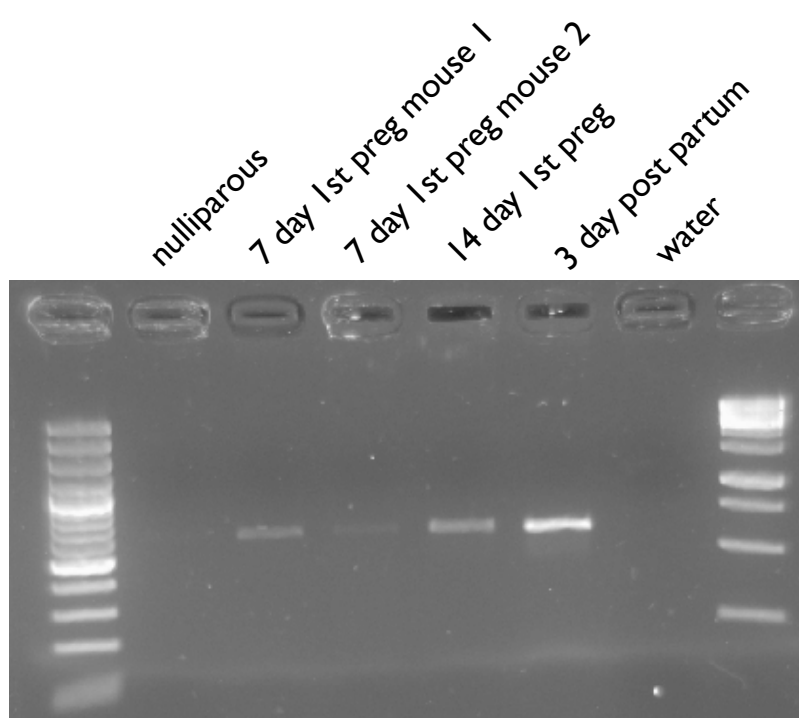

Supplement: Additional file 3 — Recombination of Rosa-lsl-YFP locus in pregnant WapCre;Rosa-lsl-YFP glands. Genomic DNA was harvested from mammary epithelial organoids from double transgenic mice (WapCre;Rosa-lsl-YFP) at the indicated developmental time points. Polymerase chain reaction (PCR) primers flanking the loxP sites of the Rosa26-lox-Stop-lox-YFP locus amplify a 578-bp band only following Cre-mediated recombination. The excision of the stop sequence was detectable at 7 days of the first pregnancy, although the extent of recombination was variable between animals. Rosa-lsl-YFP, Rosa26-lox-Stop-lox-yellow fluorescent protein. [file bcr3593-S3.pdf]

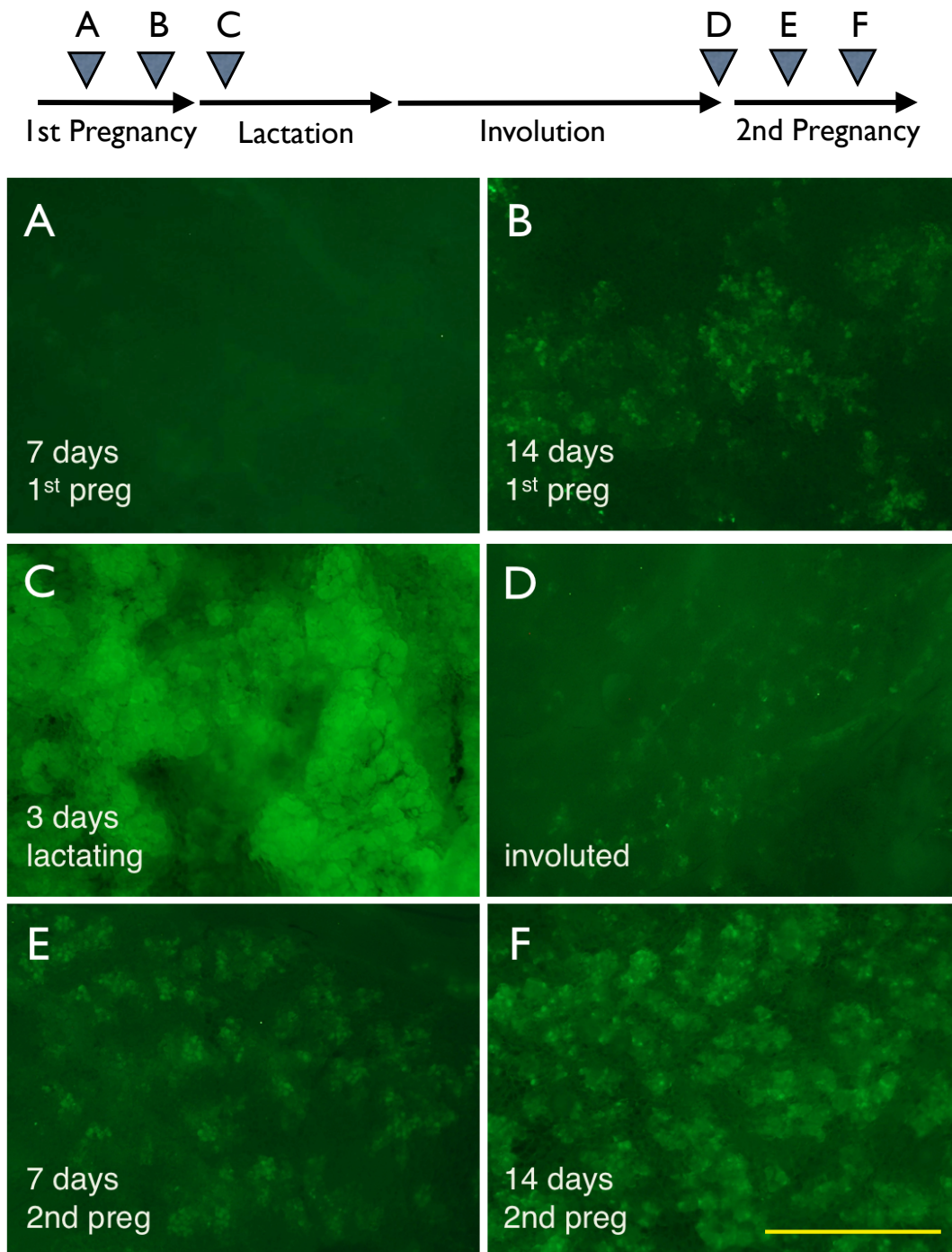

Supplement: Additional file 4 — Analysis of parity-identified mammary epithelial cell (PI-MEC) labeling in sequential pregnancies by wholemount stereoscopy. Mouse thoracic mammary glands were dissected from WAP-Cre;Rosa-lsl-YFP double transgenic mice at the following stages: 7 days (A) and 14 days (B) of the first pregnancy, 3 days of lactation (C), involuted (>6 weeks after weaning) (D), and 7 days (E) and 14 days (F) of the second pregnancy. Yellow fluorescent protein (YFP) fluorescence is visualized by wholemount microscopy of unfixed mammary glands. Bar is 1 mm. [file bcr3593-S4.pdf]

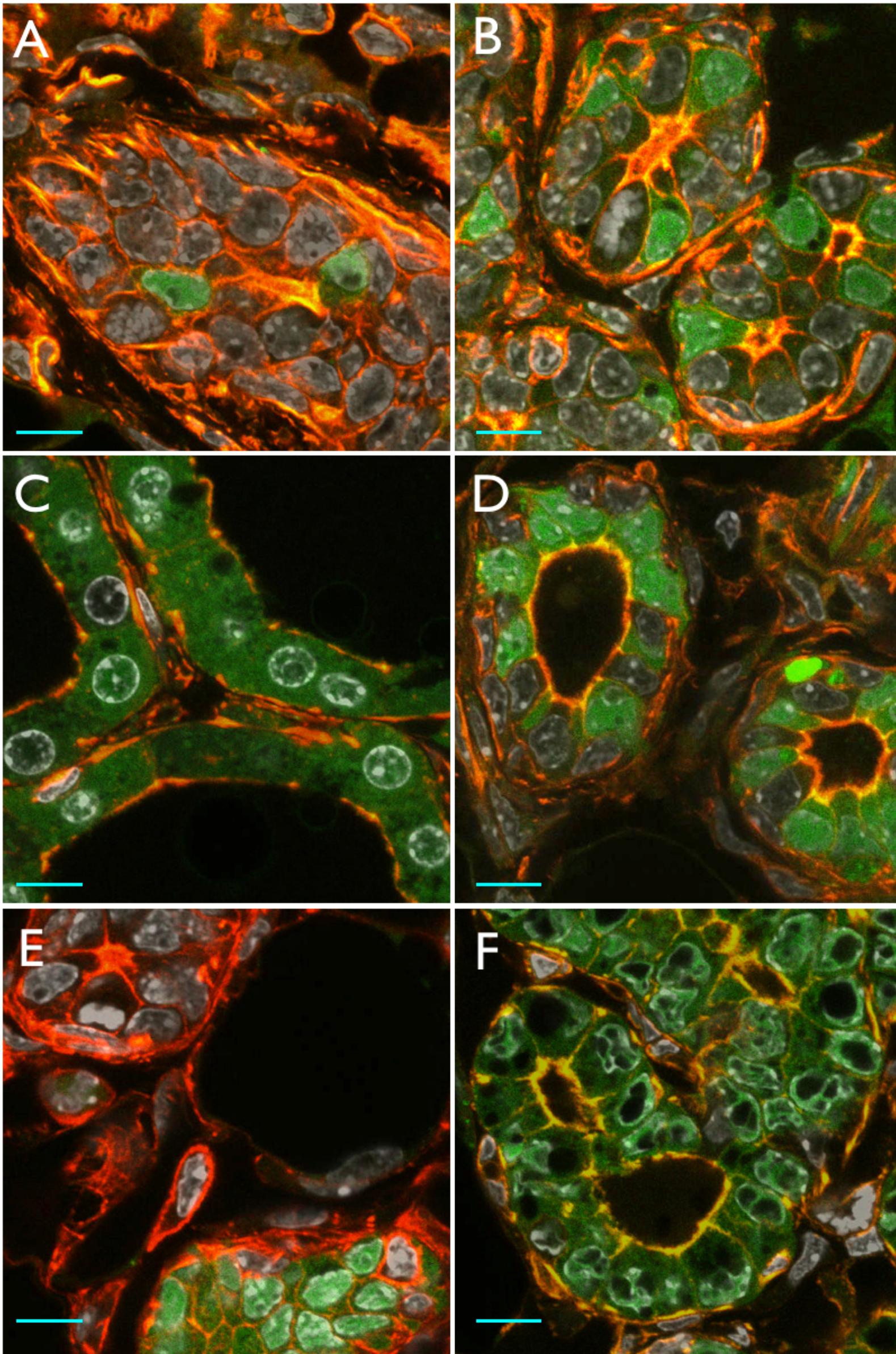

Supplement: Additional file 5 — Parity-identified mammary epithelial cell (PI-MEC) cryosections close up. Close-up views of the high-magnification confocal images from Figure 2, showing yellow fluorescent protein (YFP) fluorescence (green) from cryosections counterstained with rhodamine-conjugated phalloidin (red) and 4′,6-diamidino-2-phenylindole (DAPI) (grey). WAP-Cre;Rosa-lsl-YFP mouse mammary glands were harvested at 7 days (A) and 14 days (B) of the first pregnancy, at 3 days of lactation (C), at 6 weeks post-involution (D), and at 7 days (E) and 14 days (F) of the second pregnancy. Scale bar is 10 μm. Rosa-lsl-YFP, Rosa26-lox-Stop-lox-yellow fluorescent protein. [file bcr3593-S5.pdf]

A

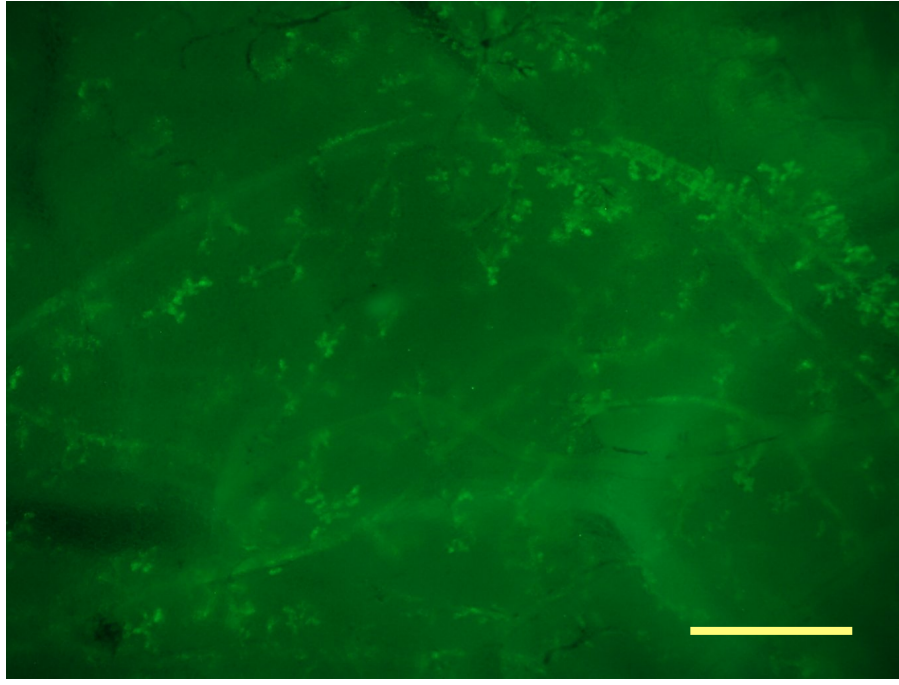

B

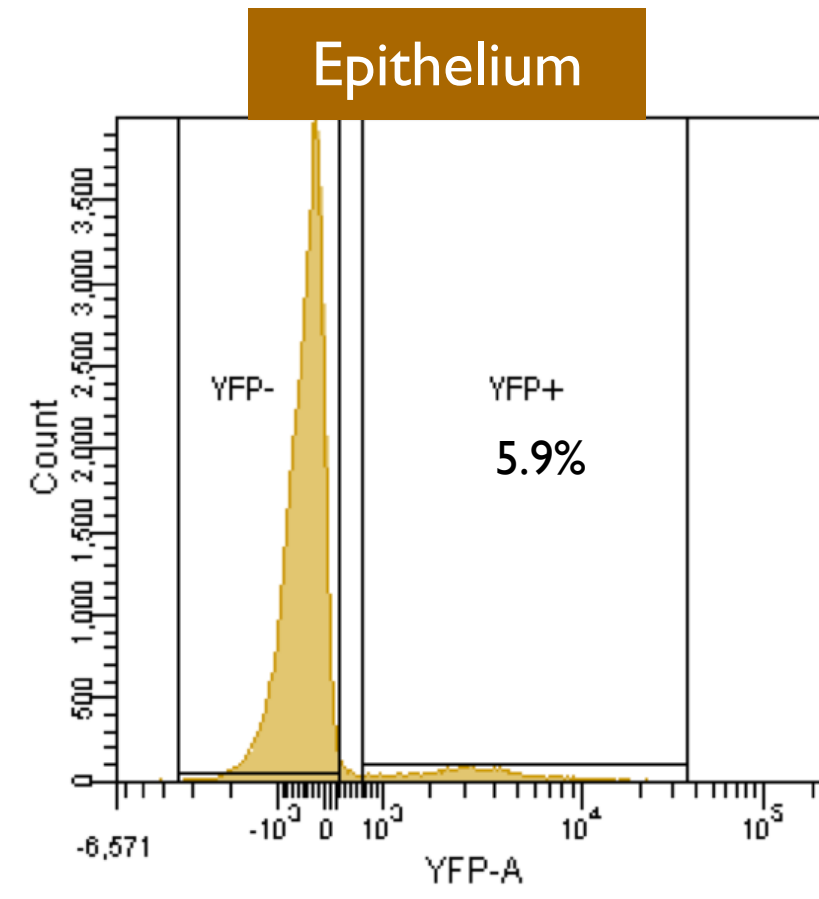

Supplement: Additional file 6 — Day 7 first pregnant mammary gland with elevated parity-identified mammary epithelial cell (PI-MEC) labeling. One day 7 first pregnant WapCre; RosaYFP mouse out of five displayed extensive recombination and yellow fluorescent protein (YFP) expression throughout ductal and alveolar network, seen by (A) wholemount fluorescence of a thoracic gland and (B) fluorescence-activated cell sorting (FACS) histogram of YFP fluorescence of mammary epithelial cells from pooled abdominal and inguinal glands. Bar is 1 mm. [file bcr3593-S6.pdf]

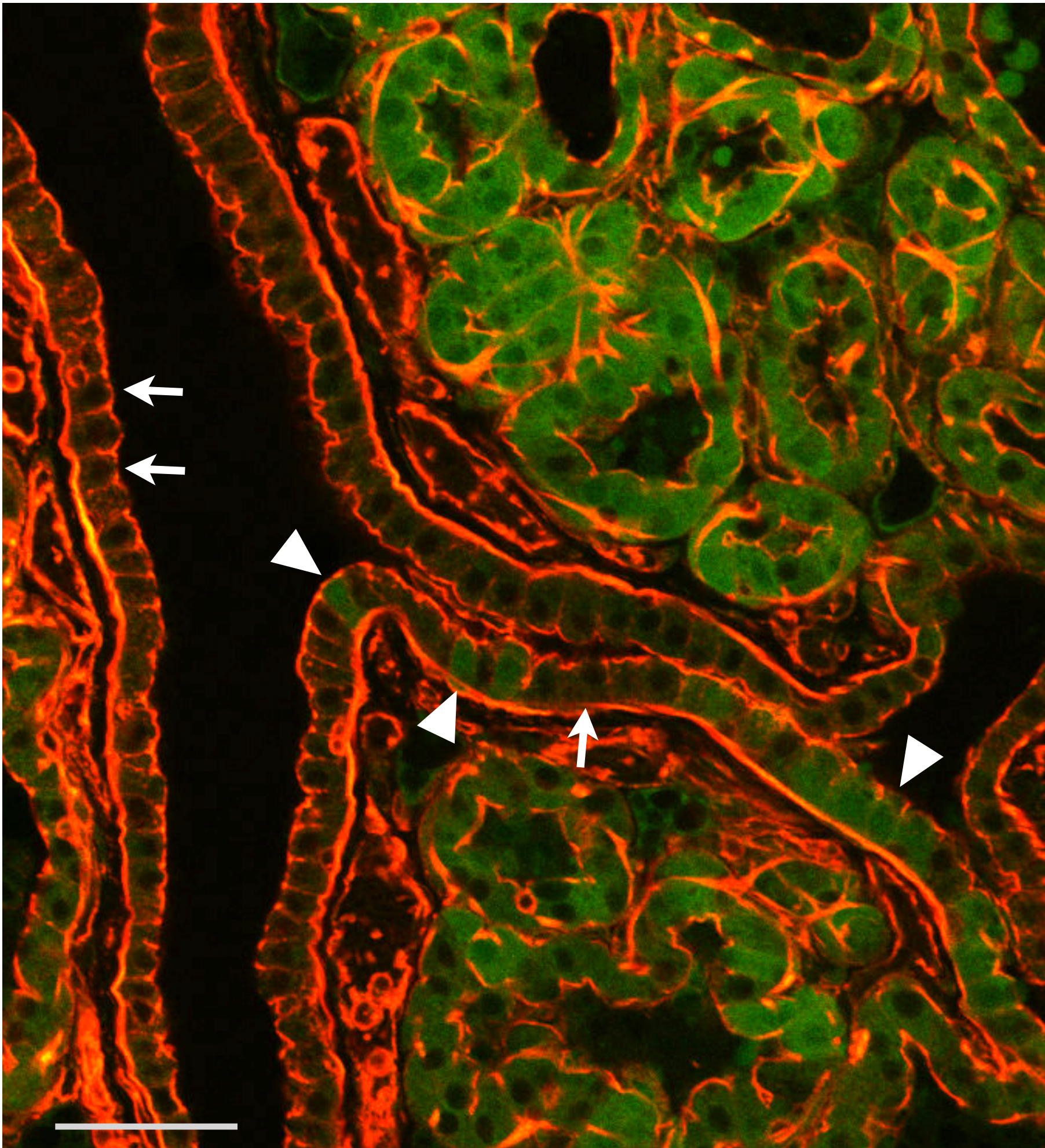

Supplement: Additional file 7 — Ductal labeling in a lactating gland. Enlarged section of top panel of Figure 2C. Cryosection of a mammary gland obtained 3 days post-partum from a WAP-Cre;Rosa-lsl-YFP mouse showing yellow fluorescent protein (YFP) expression (green) and counterstained with rhodamine-labeled phalloidin (red). Representative confocal microscope image captured with a 20× objective lens. Arrows point to unlabeled ductal cells and arrowheads to YFP-labeled ductal cells. Scale bar is 50 μm. [file bcr3593-S7.pdf]

Alveolar genes

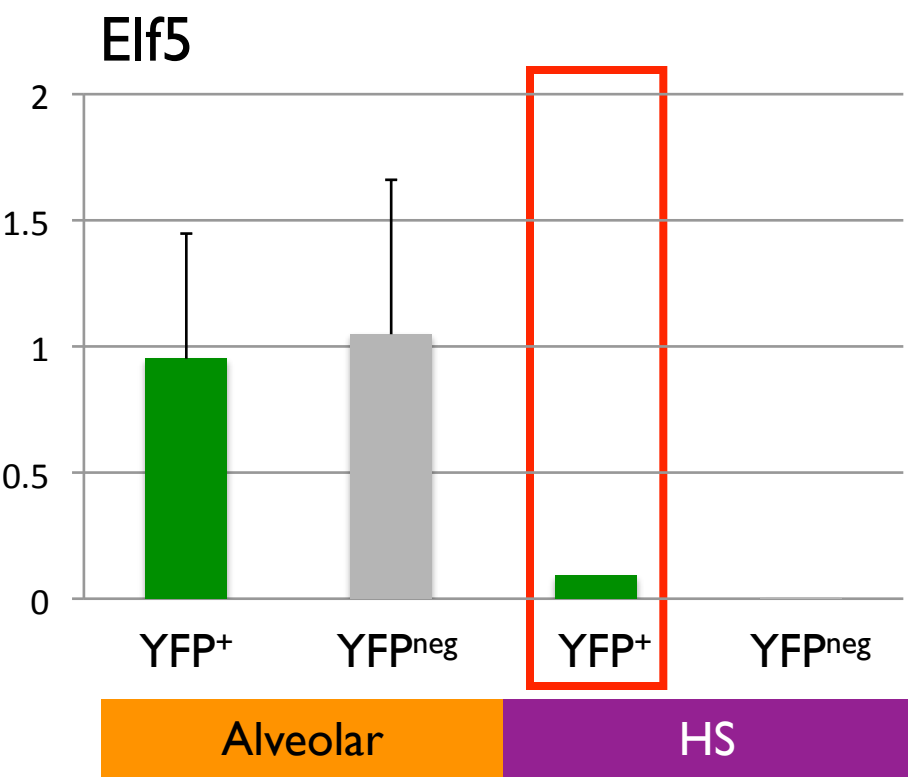

Hormone sensing genes

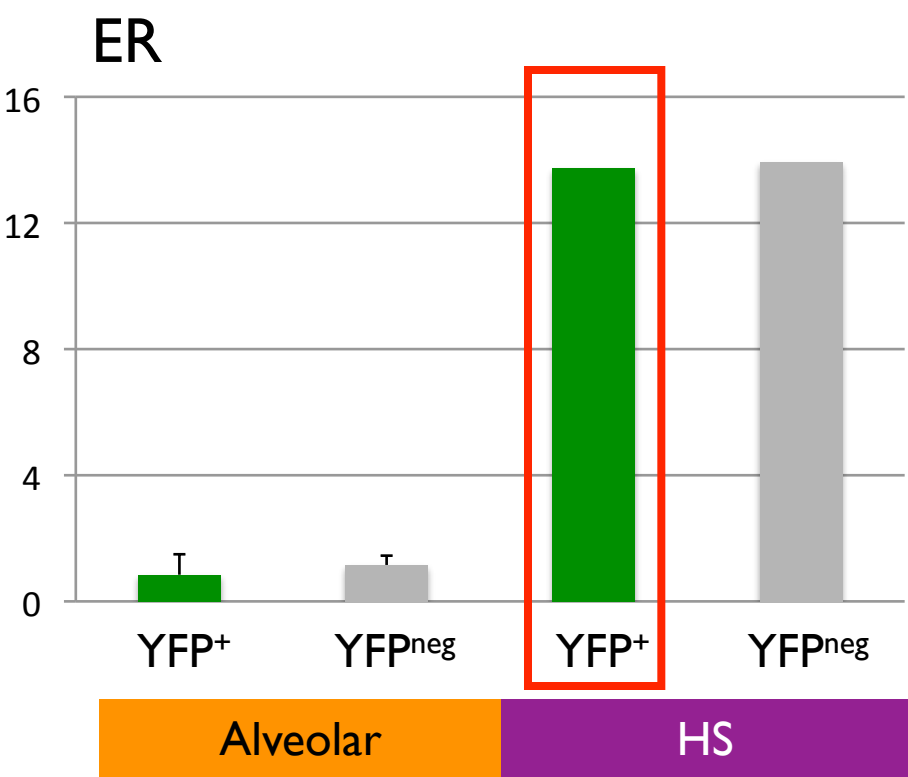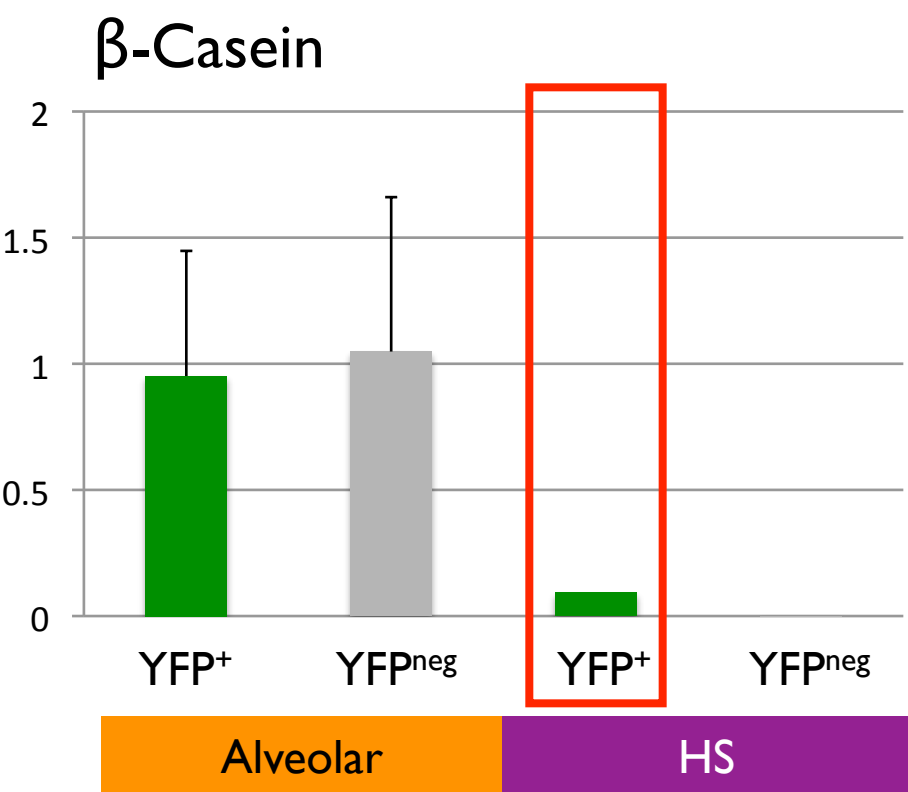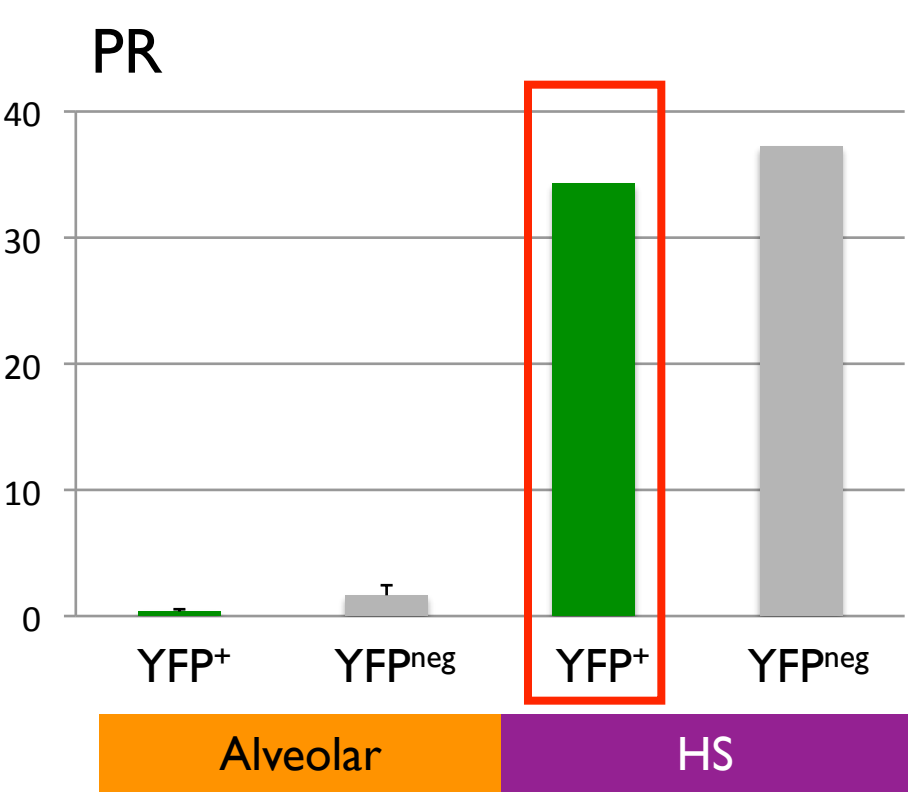

Supplement: Additional file 8 — Quantitative polymerase chain reaction (qPCR) identity of estrogen receptor-positive/yellow fluorescent protein-positive (ER+/YFP+) double-positive cells in primiparous mammary epithelial cells (MECs). Luminal single cells were isolated from parous WapCre;Rosa-lsl-YFP mammary glands and sorted directly into lysis buffer before reverse transcriptase (RT) and qPCR. Rare YFPpos hormone-sensing (HS) (Sca1hi CD49blo) cells (boxed in red) express similar levels of ERα and progesterone receptor (PR) as YFPneg HS cells and do not express alveolar marker genes Elf5 or β-Casein. Error bars reflect standard deviation (SD) for alveolar samples of 500 cells from three individual mice. Note that the HS data represent one tube of 500 cells from a pooled sample of five mice. [file bcr3593-S8.pdf]

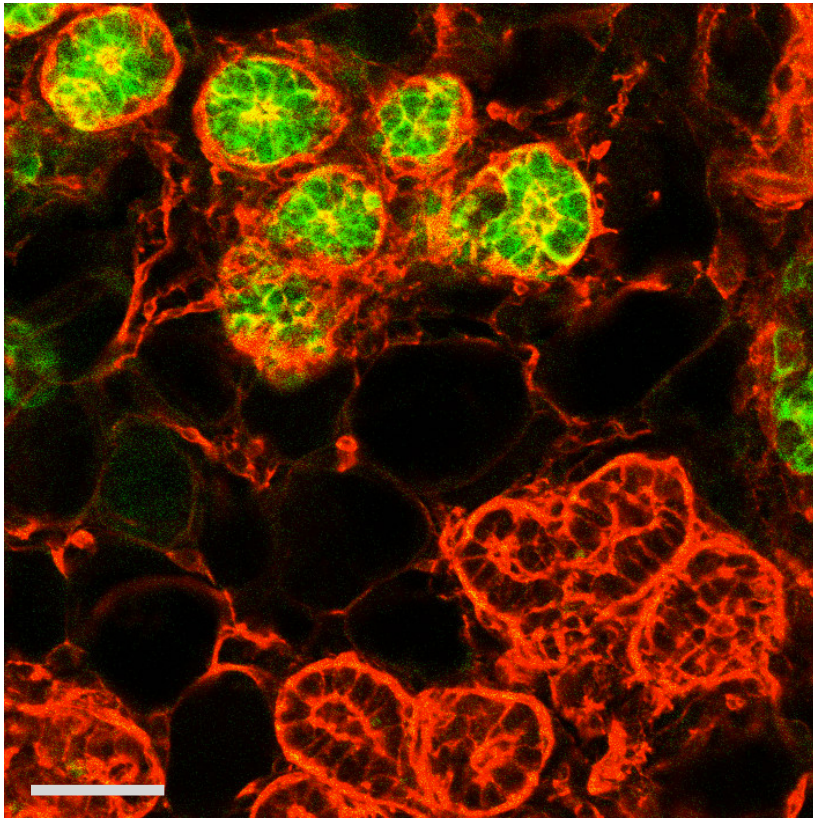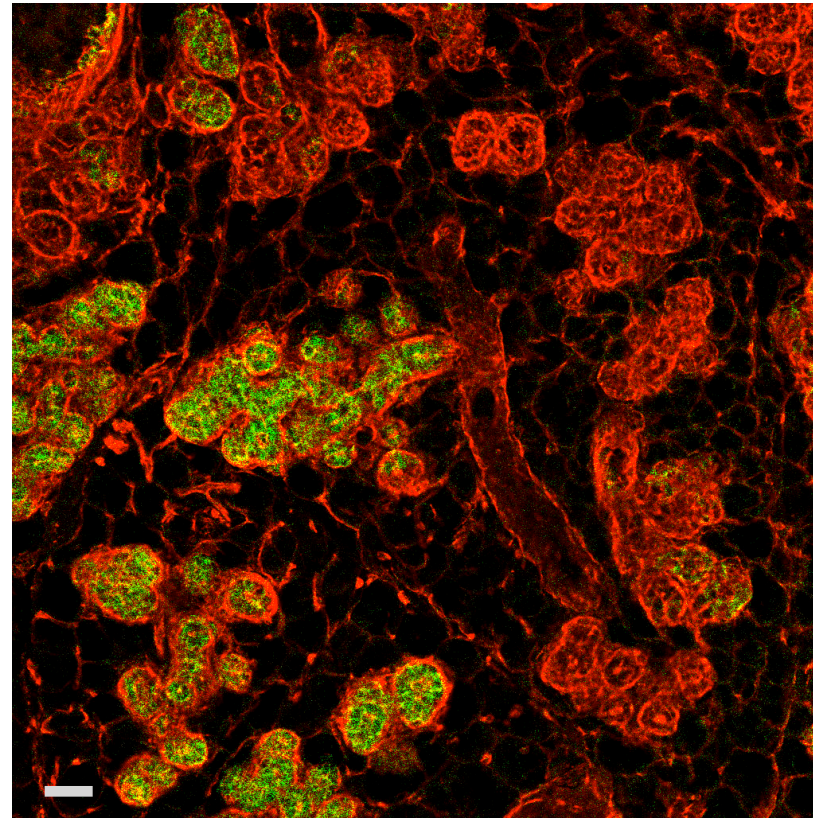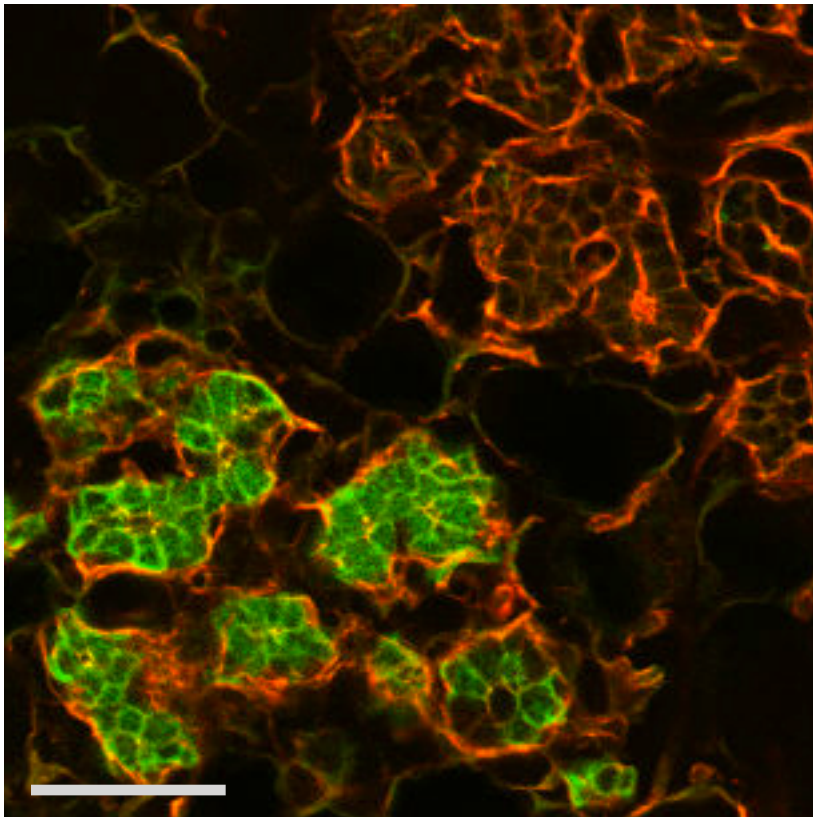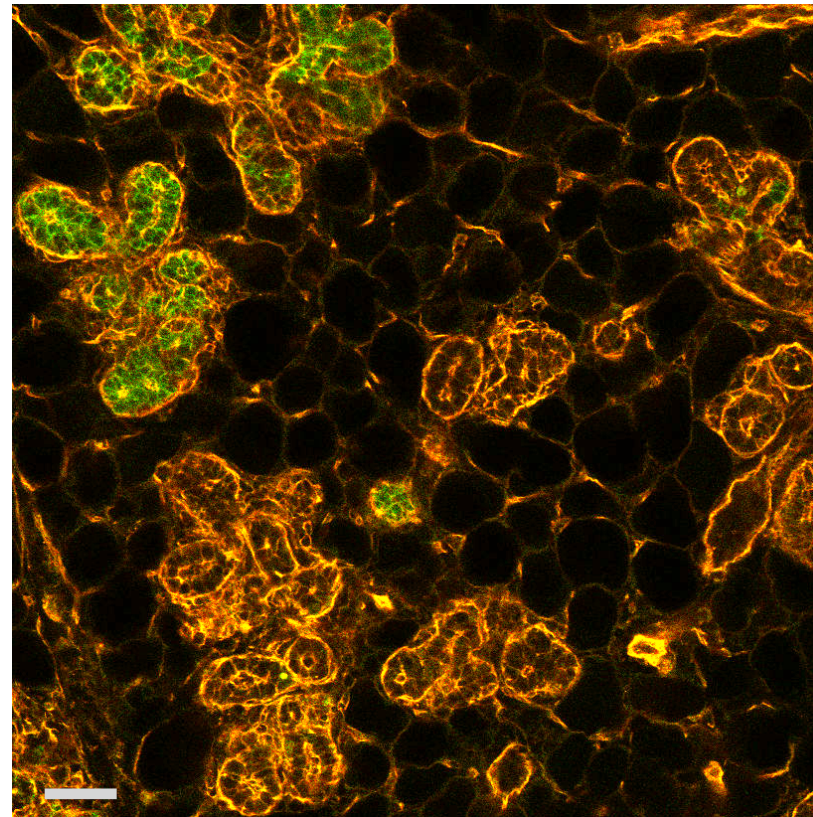

Supplement: Additional file 9 — Contribution of parity-identified mammary epithelial cells (PI-MECs) to alveologenesis occurs in clusters. Cryosections of mammary glands from WAP-Cre;Rosa-lsl-YFP mice at day 7 of the second pregnancy showing yellow fluorescent protein (YFP) expression (green) counterstained with rhodamine-conjugated phalloidin (red). Grey bar is 50 μm. [file bcr3593-S9.pdf]

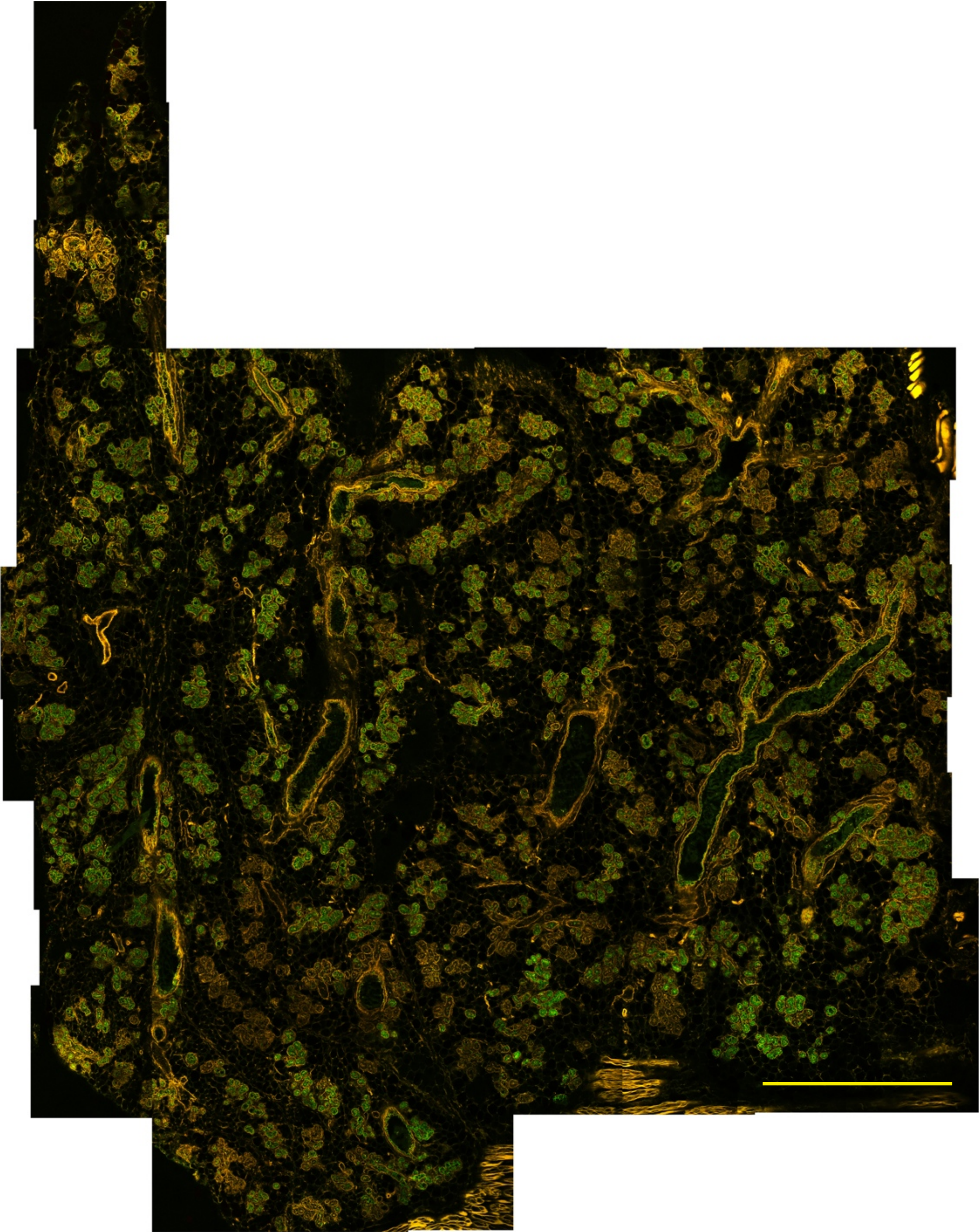

Supplement: Additional file 10 — Alveolar section map of a mammary gland at day 7 of the second pregnancy. A cryosection of a thoracic mammary gland was stained with rhodamine-conjugated phalloidin, imaged by confocal microscopy in overlapping fields covering all alveoli within the section, assembled into a contiguous map with Adobe Photoshop (Adobe Systems, Mountain View, CA, USA), and scored for yellow fluorescent protein (YFP) expression per alveolus. This mouse mammary gland represents Figure 5B. Bar is 1 mm. [file bcr3593-S10.pdf]

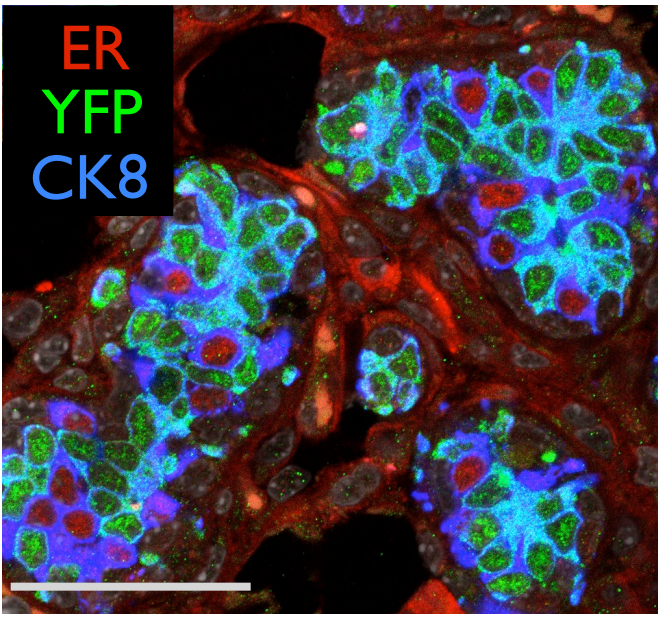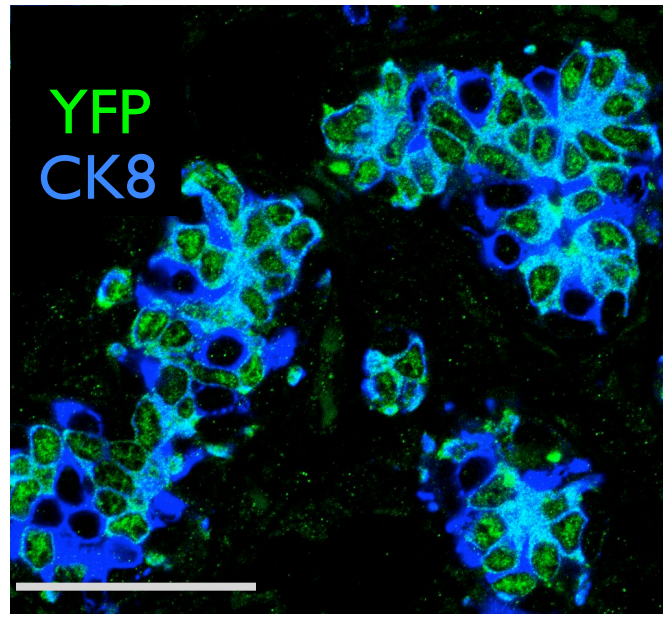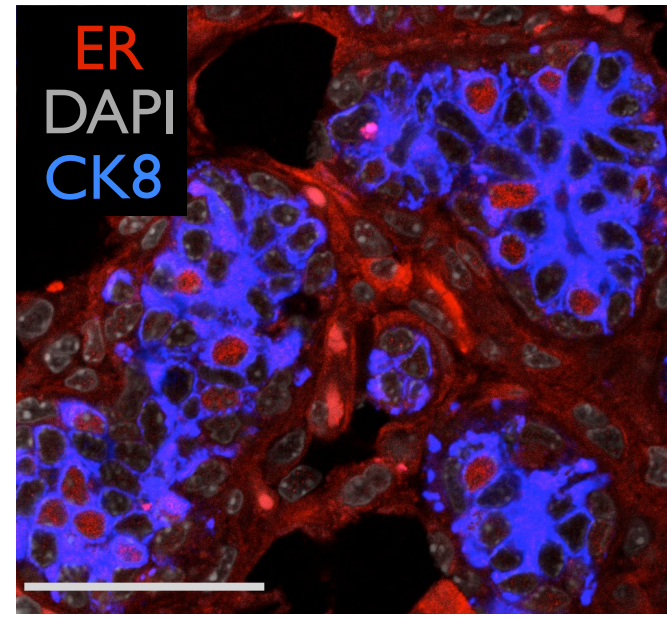

Supplement: Additional file 12 — Parity-identified mammary epithelial cells (PI-MECs) generally do not generate hormone-sensing cells. Representative confocal image of immunofluorescent staining of a paraffin section from a day 7 second pregnancy gland showing a region where all luminal (cytokeratin-8-positive, or CK8+, blue) estrogen receptor (ER)-negative cells are derived from PI-MECs (green). In the same alveoli, the luminal (CK8+, blue) cells that express the ER (ER+, red) are all yellow fluorescent protein-negative (YFPneg). Scale bar is 10 μm. [file bcr3593-S12.pdf]
