# Supplementary material for: New insights into lineage restriction of mammary gland epithelium using parity-identified mammary epithelial cells
Source: Breast Cancer Res. 2014 Jan 7;16(1):R1. doi: 10.1186/bcr3593 (PMC3978646; doi:10.1186/bcr3593)

A

Mouse	YFP neg alveoli	%YFP neg	partial YFP pos alveoli	%YFP partial	YFP pos alveoli	%YFP pos	Total alveoli in section
A	714	93%	18	2%	34	4%	766
B	290	15%	132	7%	1564	79%	1986
C	2447	83%	125	4%	375	13%	2947
X	597	49%	569	46%	65	5%	1231
Y	3127	61%	1216	24%	820	16%	5163
Z	2248	49%	1877	41%	451	10%	4576

B

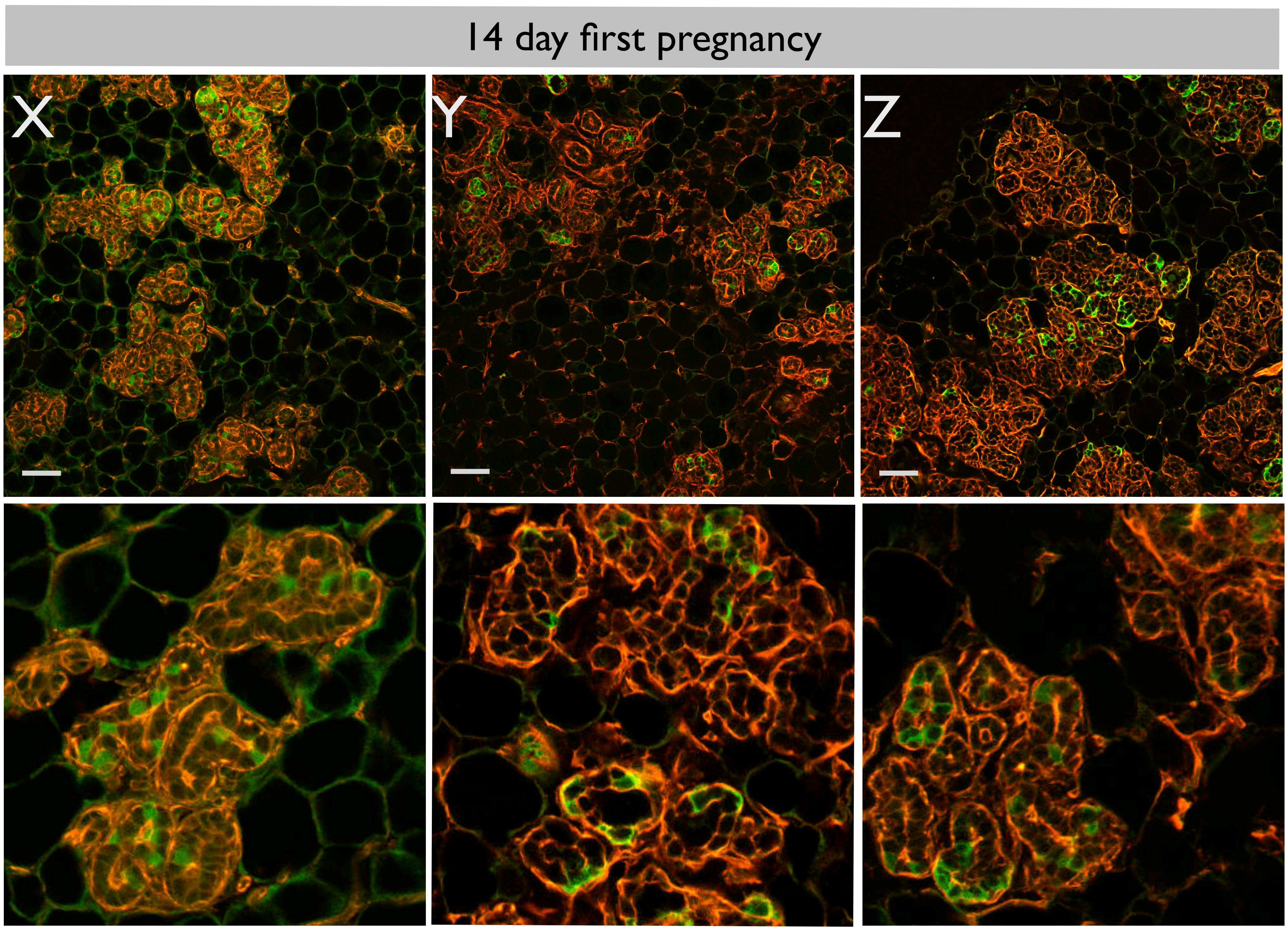

Supplement: Additional file 11 — Quantification of yellow fluorescent protein (YFP)-positive alveoli. (A) Summary of tabulated raw alveoli counts for mice scored in Figure 5D (at day 7 of the second pregnancy, A-B) and for three mice at day 14 of their first pregnancy (X-Z, Figure 5E). (B) Confocal images for mice X-Z showing the mainly partial labeling of alveoli at this stage. Scale bar is 50 μm. [file bcr3593-S11.pdf]
